# Mechanism of biomolecular recognition of trimethyllysine by the fluorinated aromatic cage of KDM5A PHD3 finger

**DOI:** 10.1038/s42004-020-0313-2

**Published:** 2020-06-01

**Authors:** Bas J. G. E. Pieters, Maud H. M. Wuts, Jordi Poater, Kiran Kumar, Paul B. White, Jos J. A. G. Kamps, Woody Sherman, Ger J. M. Pruijn, Robert S. Paton, Thijs Beuming, F. Matthias Bickelhaupt, Jasmin Mecinović

**Affiliations:** 1 Institute for Molecules and Materials, Radboud University, Heyendaalseweg 135, 6525 AJ Nijmegen, The Netherlands; 2 ICREA and Departament de Química Inorgànica i Orgànica & IQTCUB, Universitat de Barcelona, Martí i Franquès 1-11, 08028 Barcelona, Spain; 3 Chemistry Research Laboratory, University of Oxford, 12 Mansfield Road, OX1 3TA Oxford, UK; 4 Schrӧdinger, Inc., 120 West 45th Street, New York, NY 10036 USA; 5 Silicon Therapeutics, 451 D St., Boston, MA 02210 USA; 6 Radboud Institute for Molecular Life Sciences, Radboud University, Geert Grooteplein Zuid 26-28, 6525 GA Nijmegen, The Netherlands; 7 Department of Theoretical Chemistry and Amsterdam Center for Multiscale Modeling, Vrije Universiteit Amsterdam, De Boelelaan 1083, 1081 HV Amsterdam, The Netherlands; 8 Department of Physics, Chemistry and Pharmacy, University of Southern Denmark, Campusvej 55, 5230 Odense, Denmark; 9 Present Address: Latham Biopharm Group 101 Main Street, Suite 1400 Cambridge, MA, 02142 USA

**Keywords:** Chemical tools, Post-translational modifications, Computational chemistry, Methylation

## Abstract

The understanding of biomolecular recognition of posttranslationally modified histone proteins is centrally important to the histone code hypothesis. Despite extensive binding and structural studies on the readout of histones, the molecular language by which posttranslational modifications on histone proteins are read remains poorly understood. Here we report physical-organic chemistry studies on the recognition of the positively charged trimethyllysine by the electron-rich aromatic cage containing PHD3 finger of KDM5A. The aromatic character of two tryptophan residues that solely constitute the aromatic cage of KDM5A was fine-tuned by the incorporation of fluorine substituents. Our thermodynamic analyses reveal that the wild-type and fluorinated KDM5A PHD3 fingers associate equally well with trimethyllysine. This work demonstrates that the biomolecular recognition of trimethyllysine by fluorinated aromatic cages is associated with weaker cation–π interactions that are compensated by the energetically more favourable trimethyllysine-mediated release of high-energy water molecules that occupy the aromatic cage.

## Abstract (web summary)

Lysine trimethylation is a key post-translational modification, which some epigenetic reader proteins detect through binding to aromatic cages involving cation-π interactions. Here systematic modification of the aromatic cage reveals that weaker cation-π interactions do not correlate with weaker binding owing to a compensating release of bound water molecules.

## Introduction

Posttranslational modifications on histone proteins have a profound effect on the structure and function of human chromatin^1–3^. Many covalent modifications have been identified and characterized on histone tails and core histones; methylation, acetylation, phosphorylation and ubiquitination have been known for some time, whereas crotonylation and succinylation, among others, have been discovered more recently^4–7^. Methylated lysine residues can exist in the form of monomethyllysine (Kme), dimethyllysine (Kme2) or trimethyllysine (Kme3), and can lead to gene activation or repression, depending on the histone site and methylation state^8^. Histone lysine methylation is dynamically regulated by three classes of functionally related proteins^9^. The installation of the methyl group(s) from *S*-adenosylmethionine (SAM) onto lysine is catalyzed by histone lysine methyltransferases (KMTs)^10^. The opposite reaction, i.e., the removal of methyl group(s) from methylated lysine, is catalyzed either by flavin-dependent lysine specific demethylases or a larger family of non-heme Fe(II) and 2-oxoglutarate (2OG)-dependent histone lysine demethylases (KDMs)^11^. Recent structural and functional studies have revealed that histones that possess unmethylated and methylated lysine residues can be specifically recognized by a large number of reader domain proteins that differ in the composition of the lysine recognition site^12,13^. Electrostatic interactions and H-bonding appear to be of central importance in the recognition of unmethylated lysines by interacting reader domains (e.g. ADD, BAH, PZP)^13^. Similarly, electrostatic interactions and H-bonding play important roles in the readout of the lower methylation states Kme and Kme2 via a cavity-insertion binding mode (e.g. by 53BP1 tandem tudor domains, MBT domains, ankyrin repeats)^13^. Numerous epigenetic reader proteins, including plant homeodomain (PHD) zinc fingers and members of the Royal superfamily (tandem tudor domain, chromodomain and PWWP domain), have been involved in the recognition of trimethyllysine via the so-called surface-groove binding mode^13^. Despite different folding patterns, these reader proteins have a common feature as they all possess electron-rich aromatic cages, most often comprised of 1–4 side chains of Phe, Tyr and Trp, although some cages also include the negatively charged Asp or Glu residues^13,14^. Comparative binding studies between trimethyllysine and its neutral carba analogue led to the conclusion that the recognition of the positively charged trimethyllysine by the aromatic cage containing readers is predominantly driven by a combination of favourable cation–π interactions and the release of high-energy water molecules located inside the aromatic cages^15–17^.

To provide a deeper understanding of the origin of molecular recognition of trimethyllysine by aromatic cages, we report here a complementary physical-organic chemistry approach in which the two electron-rich tryptophans that solely constitute the aromatic cage of KDM5A reader are substituted by fluorinated tryptophans, thus resulting in electron-poorer π systems. Our experimental and computational investigations reveal that despite weaker aromatic character of fluorinated cages, the association between trimethyllysine and fluorinated KDM5A is comparable to that of the wild-type KDM5A. The underlying molecular mechanism for such observation is a well-balanced compensation between energetically less favourable cation–π interactions and more favourable release of high-energy water molecules that occupy fluorinated aromatic cages.

## Results

### Physical-organic chemistry approach

**Fig. 1:**
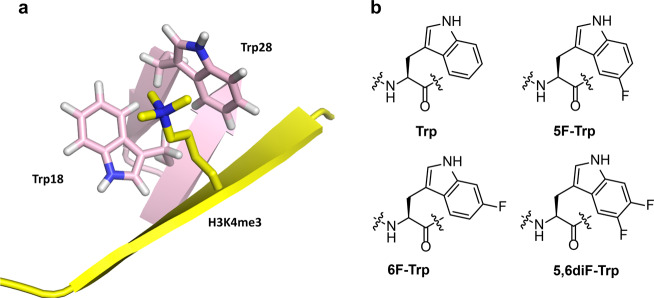
The recognition of trimethyllysine by the KDM5A PHD3 finger. **a** View from the complex of the KDM5A PHD3 finger (pink) with H3K4me3 histone peptide (yellow) (PDB: 2KGI); **b** Structures of tryptophan and fluorinated tryptophan residues for probing the strength of cation–π interactions.

The examination of cation–π interactions in biomolecular recognition of positively charged ligands using fluorinated tryptophans was pioneered by Dougherty and coworkers^18–25^. We envisioned that this elegant chemical approach could be employed for probing the involvement of cation–π interactions in the readout of trimethyllysine-containing histones by epigenetic reader domains. We chose the PHD3 finger of KDM5A reader protein as a model system, because its specific recognition of H3K4me3 is required for leukemogenesis and, importantly, its aromatic cage is composed of only two tryptophan residues (Trp18 and Trp28) (Fig. 1a)^26^. Since these are the only two tryptophans in the entire KDM5A PHD3 domain, we envisioned that an auxotrophic *E. coli* strain could be used to specifically incorporate fluorinated tryptophan residues directly into its aromatic cage. The presence of only two tryptophans eliminates the risk for perturbation of the reader domain structure as a result of additional fluorinated tryptophan residues outside the region of interest, namely the aromatic cage. This strategy allows us to investigate the effect of the aromatic cage’s π-electrons on trimethyllysine recognition by fluorinating the indole rings of the tryptophans at position 5 (5F-Trp), position 6 (6F-Trp), and at positions 5 and 6 (5,6diF-Trp) (Fig. 1b). Fluorination of tryptophan residues was ideal due to (i) fluorine’s electronegativity that can be exploited to reduce the electron density of tryptophan’s indole rings, and (ii) the comparable size of fluorine and hydrogen, allowing for minimal structural perturbations of the protein. Although it is presently unclear how the fluorination of the aromatic cage affects the energetics of water molecules that occupy such cages, our physical-organic approach also takes into consideration the role of water in the readout of trimethyllysine by the KDM5A PHD3 finger.

### Biochemical and biophysical studies of fluorinated KDM5A

**Fig. 2:**
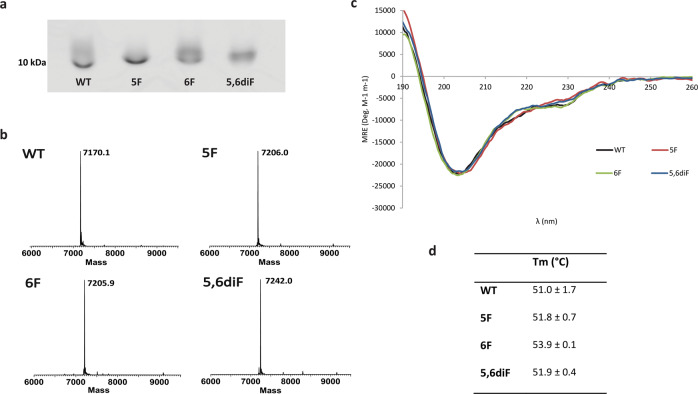
Biochemical and biophysical analyses of wild-type and fluorinated KDM5A PHD3 fingers. **a** 15% Tris-Tricine SDS-PAGE showing the purified, untagged KDM5A PHD3 fingers. From left to right: Wild-type KDM5A, 5F-KDM5A, 6F-KDM5A and 5,6diF-KDM5A. **b** ESI-MS data showing the masses of the purified KDM5A PHD3 fingers. The wild-type protein has a mass of 7170.1 Da, whereas the 5F and 6F alloproteins show a mass increase of +35.9 and +35.8 Da, respectively, and 5,6diF shows an increase of +71.9 Da. **c** CD spectra showing the purified PHD3 fingers over a range of 190–260 nm. Black: Wild-type KDM5A, red: 5F-KDM5A, green: 6F-KDM5A and blue: 5,6diF-KDM5A. **d** Melting temperatures of KDM5A PHD3 fingers obtained by DSF.

The KDM5A PHD3 finger was expressed in the auxotrophic, tryptophan deficient, *E. coli* Castellani and Chalmers strain in a method similar to the procedure described by Budisa and coworkers^27,28^. We successfully produced the protein variants with the three tryptophan analogues: 5F-Trp, 6F-Trp and 5,6diF-Trp in the KDM5A PHD3 reader domain. Additional attempts to incorporate 4,5,6,7tetraF-Trp, the most electron-poor tryptophan analogue, did not lead to production of detectable amounts of fluorinated KDM5A. Interestingly, the wild-type (WT) construct expressed significantly less well than its fluorinated counterparts. Wild-type and fluorinated KDM5A PHD3 fingers were purified using standard biochemical techniques to obtain proteins of high purity in reasonable yields (Fig. 2a and Supplementary Fig. 1). The incorporation of fluorinated tryptophans into KDM5A PHD3 was further verified by denaturing ESI-MS analyses (Fig. 2b). ESI mass spectra confirmed that the wild-type KDM5A PHD3 domain indeed had a mass of 7170.1 Da, and that the incorporation of fluorinated tryptophan residues led to the expected mass increase of 18 Da per fluorine (7206.0, 7205.9 and 7242.0 Da for 5F-KDM5A, 6F-KDM5A and 5,6diF-KDM5A, respectively). Additionally, circular dichroism (CD) spectra indicated that structures of the proteins containing the various fluorinated tryptophan analogues are identical to that of the wild-type protein (Fig. 2c). These results led us to conclude that the KDM5A alloproteins all have similar foldings and that no structural perturbations have been introduced by incorporating fluorinated tryptophans. This finding was further supported by the fact that differential scanning fluorimetry (DSF) experiments showed no decrease in the alloprotein’s melting temperature when compared with the wild-type protein. Measured melting temperatures were 51.0 ± 1.7 °C for WT KDM5A; 51.8 ± 0.7 °C for 5F-KDM5A; 53.9 ± 0.1 °C for 6F-KDM5A and 51.9 ± 0.4 °C for 5,6diF-KDM5A (Fig. 2d). Despite the fact that the WT protein expressed markedly less well in the auxotrophic strain when compared with its fluorinated counterparts, CD and ESI-MS experiments conducted on the WT protein expressed in both *E. coli* Rosetta BL21 (DE3)pLysS (hereafter referred to as BL21) and *E. coli* Castellani and Chalmers (hereafter referred to as AUX) showed that both expression strains produced proteins with identical masses and tertiary structures (Supplementary Figs. 2 and 3). It can therefore be concluded that both expression systems produce the WT KDM5A PHD3 finger with identical structural properties.

### Thermodynamic analyses of KDM5A–H3K4me3 association

After characterizing the KDM5A proteins, the effect of fluorination of the PHD3 finger of KDM5A on binding to the H3K4me3 peptide (sequence: ARTKme3QTARKS) was examined by isothermal titration calorimetry (ITC), which provided values of the Gibbs free energy of binding (Δ*G*°), enthalpy of binding (Δ*H*°) and entropy of binding (Δ*S*°) (Table 1 and Supplementary Fig. 4). First, a comparison was made between WT protein expressed in the AUX strain and WT protein expressed in the BL21 strain, which had also been used for KDM5A expression in our previous studies^16,29^. The observed binding affinities were indistinguishable, with a *K*_d_ of 54 ± 6 nM for BL21 WT-KDM5A and 48 ± 8 nM for AUX WT-KDM5A (Table 1). Notably, thermodynamic parameters Δ*G*° (BL21-KDM5A: −9.9 ± 0.1 kcal mol^−1^ vs AUX-KDM5A: −10.0 ± 0.1 kcal mol^−1^), Δ*H*° (BL21-KDM5A: −11.6 ± 0.1 kcal mol^−1^ vs AUX-KDM5A: −11.9 ± 0.1 kcal mol^−1^) and −TΔ*S*° (BL21-KDM5A: 1.7 ± 0.2 kcal mol^−1^ vs AUX-KDM5A: 1.9 ± 0.1 kcal mol^−1^) were also observed to be virtually indistinguishable (Table 1). In conjunction with CD and ESI-MS data, these results imply that both WT proteins are identical with respect to their biophysical and binding properties.

**Table 1:** Thermodynamic data for binding of H3K4me3 to KDM5A PHD3.

	*K*_d_ (nM)	Δ*G*° (kcal mol^−1^)	Δ*H*° (kcal mol^−1^)	−TΔ*S*° (kcal mol^−1^)
BL21 WT-KDM5A	54 ± 6	−9.9 ± 0.1	−11.6 ± 0.1	1.7 ± 0.2
AUX WT-KDM5A^a^	48 ± 8	−10.0 ± 0.1	−11.9 ± 0.1	1.9 ± 0.1
5F-KDM5A	49 ± 12	−10.0 ± 0.1	−10.9 ± 0.1	0.9 ± 0.3
6F-KDM5A	57 ± 17	−9.9 ± 0.2	−12.1 ± 0.1	2.2 ± 0.3
5,6diF-KDM5A	70 ± 15	−9.8 ± 0.1	−11.7 ± 0.2	1.9 ± 0.3

10-mer H3K4me3 peptide (ARTKme3QTARS) was used. Values obtained from 7–9 repeated ITC experiments. BL21 and AUX indicate the expression system used for WT protein expression with BL21 corresponding to the Rosetta BL21 (DE3) pLysS and AUX to the Castellani and Chalmers auxotrophic strains, respectively. The stoichiometry (H3K4me3:KDM5A, *n*) = 0.97–1.03.

^a^Measured in replicate.

**Fig. 3:**
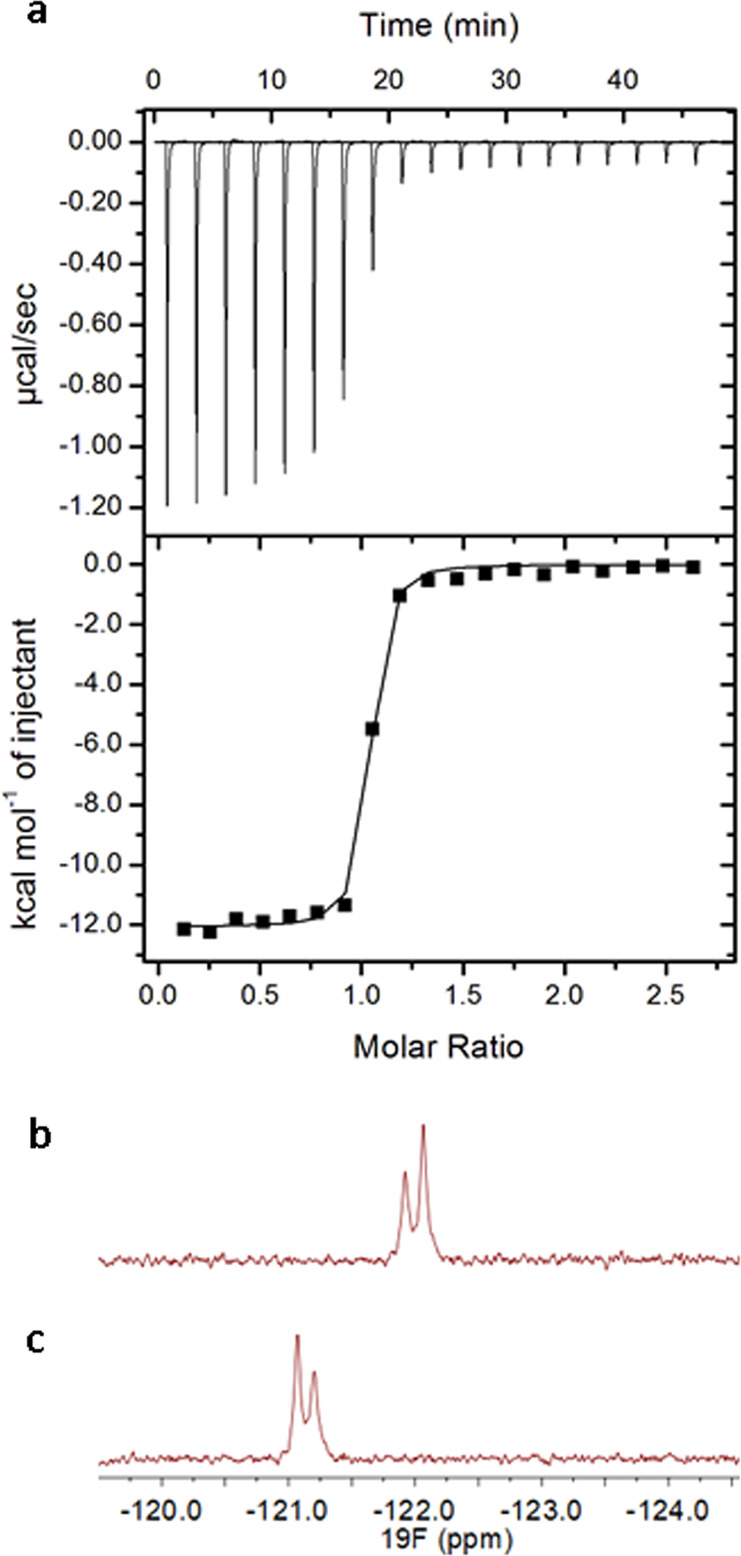
Binding of H3K4me3 to the 6F-KDM5A PHD3 finger. **a** ITC curve of 10-mer H3K4me3 histone peptide binding to 6F-KDM5A; **b** ^19^F NMR spectrum of 6F-KDM5A; **c** ^19^F NMR spectrum of 6F-KDM5A in the presence of the H3K4me3 peptide.

Next, we carried out comparative thermodynamic analysis for binding of H3K4me3 with WT KDM5A PHD3 and its fluorinated counterparts. H3K4me3 bound to all four KDM5A reader domain variants with virtually equal binding affinity; the measured Δ*G*° values for all KDM5A–H3K4me3 systems were observed to be −9.9 ± 0.1 kcal mol^−1^ (Table 1). The examination of the enthalpic and entropic terms of Δ*G*°, furthermore, showed that although small differences between WT and fluorinated KDM5A are present, they are not significant. 5F-KDM5A showed a decreased enthalpy when compared with wild-type KDM5A, with a ΔΔ*H*° of 1.0 ± 0.1 kcal mol^−1^. The decrease in enthalpy was, however, completely compensated by an increase in entropy, with a −TΔΔ*S*° of −1.0 ± 0.3 kcal mol^−1^. Binding thermodynamics for 6F-KDM5A and wild-type KMD5A are identical within standard error (Fig. 3a, Table 1). Notably, 5,6diF-KDM5A, the most electron-poor aromatic cage in our panel of cages, displayed a very similar thermodynamic signature to wild-type KDM5A. We did not observe any significant differences in values of the free energy of binding, as well as in its enthalpic and entropic contributions (Table 1). Taken together, our thermodynamic data show that addition of electron-withdrawing fluorine substituents to the indole rings that solely constitute the KDM5A’s PHD3 aromatic cage, does not reduce the protein’s binding affinity for the positively charged trimethyllysine of H3K4me3. Based on the related examinations of cation–π interactions between ammonium cations and tryptophan residues in protein–ligand associations that display significant reduction and linear trend of binding affinity upon fluorination of tryptophan, our studies suggest that cation–π interactions are not solely responsible for binding of H3K4me3 to the KDM5A PHD3 finger, as no linear reduction in binding affinity is observed upon increased fluorination of the aromatic cage^19^. As will be discussed below, desolvation effects provide another important contribution to the overall binding process, giving an explanation for maintaining the same binding affinities.

### ^19^F NMR studies of fluorinated KDM5A

We employed ^19^F NMR spectroscopy to compare the free and H3K4me3-bound forms of 6F-KDM5A (Fig. 3b, c). The ^19^F NMR spectrum of free 6F-KDM5A showed two peaks (−121.9 ppm and −122.1 ppm), thus supporting the presence of two fluorine atoms located at 6F-Trp18 and 6F-Trp28 of the PHD3 finger of KDM5A. Upon binding of H3K4me3 to 6F-KDM5A, we observed down-field shifts of approximately +1 ppm (−121.1 ppm and −121.2 ppm), consistent with the magnitude of shifts found in other ligand binding studies utilizing 6F-Trp labelled proteins^30^. A down-field shift was also observed upon binding of H3K4me3 to 5F-KDM5A (Supplementary Fig. 5). These results indicate that the positively charged trimethyllysine moiety present in the H3K4me3 peptide interacts with the fluorinated tryptophans incorporated into the aromatic cage.

Additional CD spectroscopic analysis confirmed that 5F-KDM5A and 6F-KDM5A remained stable during the NMR measurements performed at 15 °C (Supplementary Figs. 6 and 7). Moreover, CD analyses indicated a small change in the protein’s structure upon formation of the 5F-KDM5A–H3K4me3 and 6F-KDM5A–H3K4me3 complex. A small shift in mean residual ellipticity (MRE) between 215–240 nm corresponds to a more extensive β-sheet conformation. This observation is in line with the finding that H3K4me3 peptide forms a third antiparallel β-strand when complexed with the PHD3 domain of KDM5A, as also visible in the reported KDM5A–H3K4me3 structure (Fig. 1a)^26^.

### Molecular dynamics simulations of KDM5A–H3K4me3 complexes

After experimentally determining that H3K4me3 binds to fluorinated PHD3 fingers of KDM5A, we carried out molecular dynamics (MD) simulations to examine the behaviour over time of the reader–ligand complex and effects of fluorination on key interactions for binding. Four variations of the PHD3 finger of KDM5A were simulated, including the wild-type (PDB: 2KGI) and three variants containing F-substituted Trp18-Trp28 aromatic cages: 5F-Trp18/5F-Trp28, 6F-Trp18/6F-Trp28, and 5,6diF-Trp18/5,6diF-Trp28. Adopting a recently described molecular mechanics-based approach^29,31^, the four systems were solvated in a 10 Å truncated octahedral box of TIP3P water^32^, neutralised explicitly with either sodium or chloride ions, and simulated for 100 ns using the Amberff12SB force field.

**Fig. 4:**
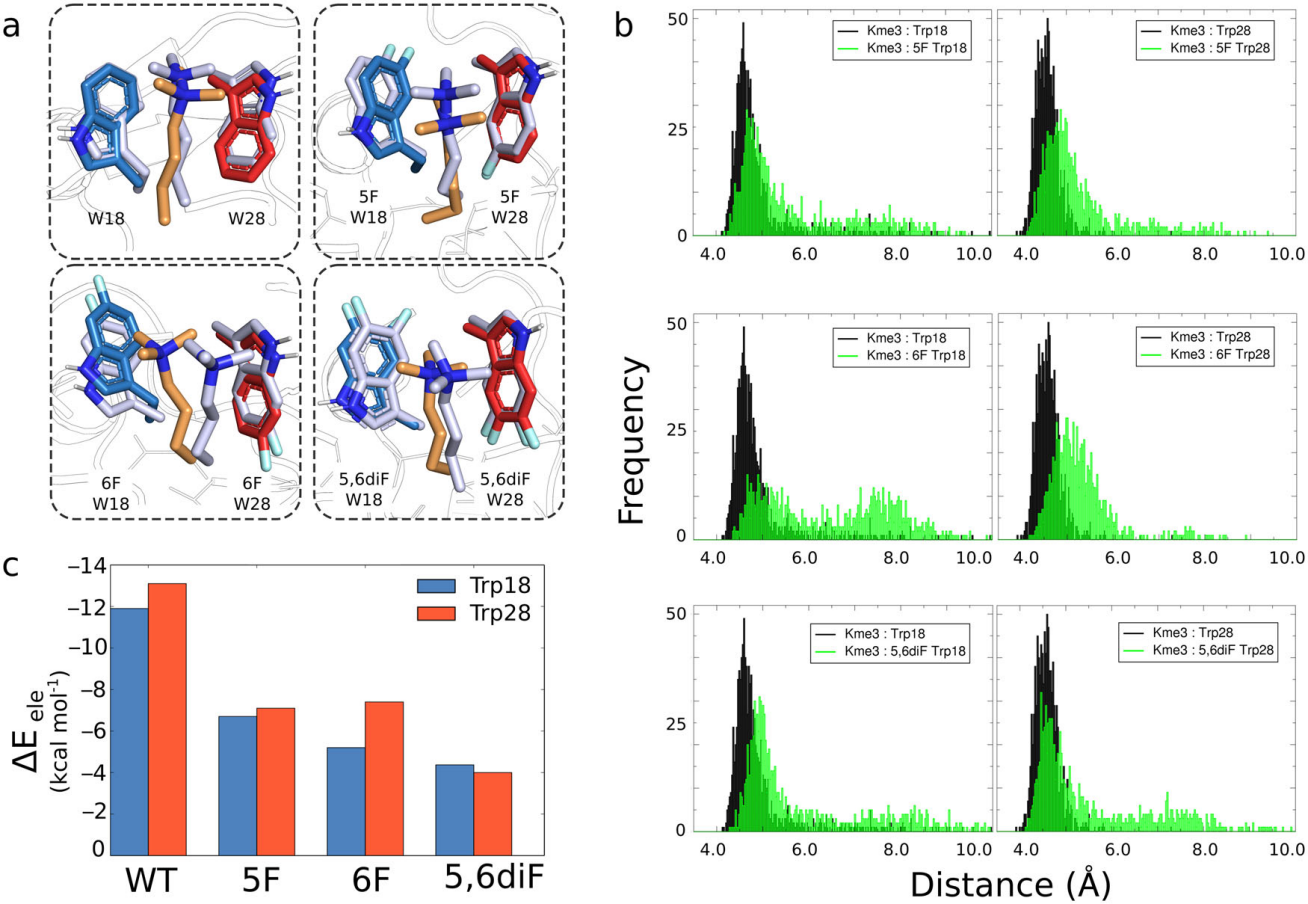
Molecular dynamics simulations of wild-type and fluorinated KDM5A PHD3 fingers with H3K4me3. **a** The active site at time 0 ns (light blue) overlaid with the structure observed at the end of the simulation at time 100 ns (H3K4me3, orange; Trp18, blue; Trp28, red) for each system. **b** Histograms of the distance between the H3K4me3 ammonium cation and each aromatic side chain in the Trp18-Trp28 cages. **c** Average Δ*E*_ele_ values of the ammonium cation of H3K4me3 to each aromatic side chain in the Trp18-Trp28 cages.

In all cases of fluorinated KDM5A, the trimethyllysine side chain of H3K4me3 occupies the aromatic cage throughout the simulation (Fig. 4a). Flexibility of the H3 chain to prioritize this interaction is demonstrated for the mutated systems (Supplementary Fig. 8). For KDM5A containing 5F-Trp18/5F-Trp28, and 5,6diF-Trp18/5,6diF-Trp28 behaviour of terminal H3 residues shows great flexibility (Supplementary Fig. 8b and d), compared with the wild-type simulation that shows little difference in the H3 backbone geometry (Supplementary Fig. 8a).

Non-covalent cation–π interactions are formed between H3K4me3 and both Trp residues, where we define this using an established geometric cutoff of 6 Å (Fig. 4b and Supplementary Figs. 9–11)^33^. To quantify the strength of these energetically favourable cation–π interactions, average Δ*E*_ele_ values were calculated between the quaternary ammonium cations of H3K4me3 to each aromatic side chain of Trp18-Trp28 (Fig. 4c and Supplementary Table 1). Effects on Δ*E*_ele_ from fluorination on the Trp side chain suggest a general trend WT > 5F ≥ 6F > 5,6diF when comparing just the indole heavy atoms or with inclusion of the electronegative fluorine substituents (Supplementary Table 2). These results indicate that the positively charged trimethyllysine predominantly interacts with the π-system of the aromatic cage, and that possible interaction with the electronegative fluorine substituents does not significantly contribute to the stabilization^34^. Fluorination results in less favourable electrostatic contributions to cation–π interactions^35^, consistent with our findings from quantum chemical studies and energy decomposition analyses (see below). Binding of H3K4me3 to 6F-KDM5A leads to differences in Δ*E*_ele_ values when comparing 6F-Trp18 and 6F-Trp28 (Supplementary Table 2) and agrees with the bimodal distribution of distance between the cation and π-face (Supplementary Fig. 8b). An overall preference for Trp28 over Trp18 is also observed for the systems, except for KDM5A containing 5,6diF-Trp where this interaction is almost equal (Fig. 4c). The stronger interaction between the quaternary ammonium cation with Trp28 has been previously observed for D-Kme3, trimethylornithine and trimethylhomolysine^29,31^. We also examined the distance calculated from the N^+^ atom of H3K4me3 to the 5- and 6-membered rings of the Trp18/Trp28, 5F-Trp18/5F-Trp28, 6F-Trp18/6F-Trp28, and 5,6diF-Trp18/5,6diF-Trp28 side chains (Supplementary Figs. 12 and 13). At time 100 ns, virtually no difference is observed when comparing the distance of the cation to the pyrrole or benzene substructure for both wild-type and F-substituted Trp18-Trp28 side chains. In line with this observation, also our quantum chemical calculations reveal only minor changes.

### Quantum chemical analyses in the gas and aqueous phase

Next, we aimed to elucidate the nature of the non-covalent interactions between the Kme3 side-chain of the histone peptide and the aromatic cage that consists of two fluorinated tryptophan residues of the KDM5A PHD3 finger (hereafter designated as TRP2 fragment) to understand the underlying origin of the recognition. We characterized quantum chemically the energetics and bonding mechanism in the four model complexes, using dispersion-corrected density functional theory at BLYP-D3BJ/TZ2P and COSMO for simulating aqueous solution, as implemented in the ADF program (Supplementary Table 3)^36^. Recently, this methodology has been successfully employed in the quantum chemical exploration and bonding analyses of trimethyllysine analogues by TRP2^16,31^. As previously discussed, from Table 2, it is seen how TRP2–Kme3 presents a bond energy of −10.2 kcal mol^−1^. This energy is almost identical with the corresponding instantaneous interaction energy Δ*E*(aq)_int_ of −10.3 kcal mol^−1^, due to an almost negligible deformation strain energy (Δ*E*(aq)_strain_ = 0.1 kcal mol^−1^) associated with the subtle variation of geometry upon complexation. The interaction energy between Kme3 and TRP2 without water is stronger (Δ*E*_int_ = −27.6 kcal mol^−1^), because the system presents an unfavourable desolvation energy of 17.3 kcal mol^−1^. The interaction energy in the absence of water, ∆*E*_int_, can be decomposed into Pauli repulsion (Δ*E*_Pauli_ = 20.8 kcal mol^−1^), electrostatic attraction (Δ*V*_elstat_ = −15.0 kcal mol^−1^), orbital interaction (Δ*E*_oi_ = −13.0 kcal mol^−1^) and dispersion (Δ*E*_disp_ = −20.4 kcal mol^−1^) terms.

**Table 2:** Quantum-chemical bonding analysis in TRP2–Kme3 systems.

	TRP2–Kme3^a^	5F-TRP2–Kme3^b^	6F-TRP2–Kme3^b^	5,6diF-TRP2–Kme3^b^
Δ*E*(aq)	−10.2	−10.4	−10.3	−10.3
Δ*E*(aq)_strain_	0.1	0.3	0.3	0.3
Δ*E*(aq)_int_	−10.3	−10.7	−10.6	−10.6
Δ*E*(desolv)_int_	17.3	14.5	14.0	11.7
Δ*E*_int_	−27.6	−25.2	−24.6	−22.3
Δ*E*_Pauli_	20.8	20.4	21.0	19.8
Δ*V*_elstat_	−15.0	−12.2	−12.0	-9.0
Δ*E*_oi_	−13.0	−12.8	−13.0	−12.6
Δ*E*_disp_	−20.4	−20.5	−20.5	−20.5
d(H_Me_-C_TRP-6MR_)	3.38	3.50	3.54	3.52
d(H_Me_-C_TRP-5MR_)	2.78	2.70	2.68	2.70

Computed at BLYP-D3BJ/TZ2P with COSMO to simulate aqueous solution. Energies in kcal mol^−1^, distances in Å. Structural rigidity imposed by the protein backbone is simulated through constrained geometry optimizations. See also Eqs. (1–3) in the ”Methods” section.

^a^TRP2 frozen, Kme3 entirely free.

^b^TRP2 frozen, α-methyl carbon fixed to position in TRP2–Kme3 optimization.

Furthermore, we performed an analogous series of analyses as the one described above, but this time for di- and tetra-fluorinated TRP2 as the aromatic cage with Kme3. First, bond energies hardly change for difluorinated 5F-TRP2–Kme3 and 6F-TRP2–Kme3, or tetrafluorinated 5,6diF-TRP2–Kme3 systems (Δ*E*(aq) = −10.3–−10.4 kcal mol^−1^). The same happens for both the deformation strain and interaction energies, with a maximum change of 0.1 kcal mol^−1^. So, even with the presence of fluorine on TRP2, complexation only very slightly changes the geometry of Kme3 side chain. However, changes appear when these interactions are analysed without water. Fluorination of TRP2 causes a weakening of the interaction in the absence of water, ∆*E*_int_, between Kme3 and TRP2 of 2.4 and 3.0 kcal mol^−1^ for 5F-TRP2–Kme3 and 6F-TRP2–Kme3, respectively, and of 5.3 kcal mol^−1^ for 5,6diF-TRP2–Kme3 (Table 2). The weakening in ∆*E*_int_ upon fluorination is countered by a less unfavourable desolvation energy. The larger desolvation energy of TRP2–Kme3 can be associated with the removal of solvent around the positive charge of the Kme3 side chain ammonium group. In the case of the fluorinated systems, obviously, the same desolvation of Kme3 still occurs. The fact that the electronegative fluorine atoms pull charge out of the aromatic rings reduces the desolvation energy of the latter, which leads to the computed overall less unfavourable Δ*E*(desolv)_int_ values.

The observation that Δ*E*_int_ in the gas phase weakens from −27.6 to −25.2, −24.6, and −22.3 kcal mol^−1^ for TRP2–Kme3, 5F-TRP2–Kme3, 6F-TRP2–Kme3, and 5,6diF-TRP2–Kme3, respectively, led us to additionally carry out the energy decomposition analysis of the interaction energy. First, it is observed that aforementioned weakening is not the result of the Pauli repulsion term, which remains quite constant among the complexes (Δ*E*_Pauli_ = 19.8–21.0 kcal mol^−1^), with a maximum difference of 1.0 kcal mol^−1^ compared with unfluorinated TRP2 system. This constant Pauli term is in agreement with the minor geometrical changes among the different systems, which can be also followed from distances enclosed in Table 2. The closest H-C distances between an NMe_3_^+^ H atom and a C atom of a tryptophan in TRP2–Kme3 is 2.78 Å, while the same H atom is 3.38 Å away from the closest C atom of the other tryptophan (Supplementary Fig. 14). For the fluorinated systems, the former distance is slightly shortened (2.68–2.70 Å), whereas the latter is lengthened (3.50–3.54 Å). Distances between the quaternary N atom of Kme3 and the centroids of the five- and six-membered rings of TRP2 can be found in Supplementary Table 4.

**Fig. 5:**
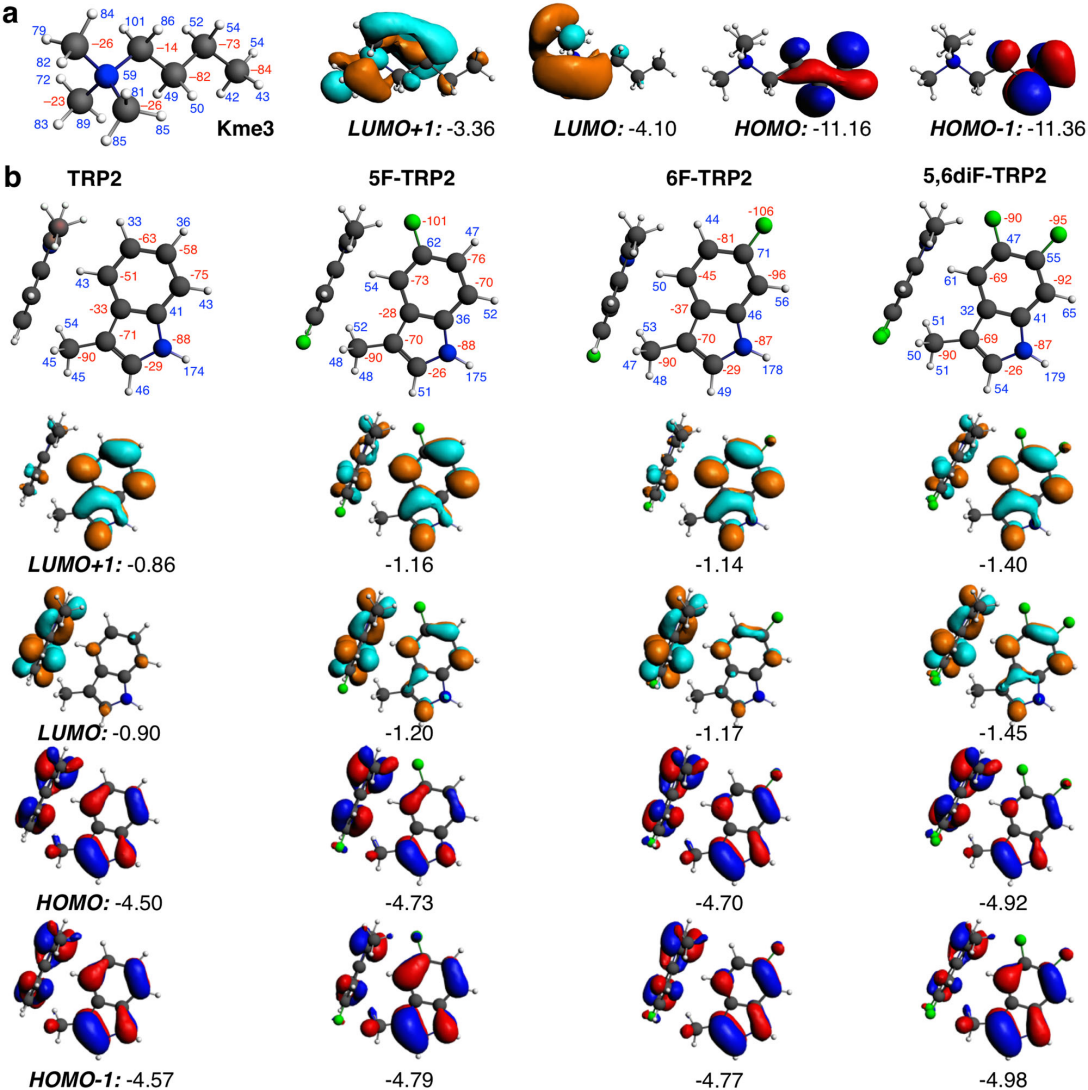
Quantum chemical analysis of TRP2–Kme3 interactions. VDD atomic charges (in mili-a.u., red = negative, blue = positive), and frontier orbitals (with orbital energies in eV, isosurface drawn at 0.03) of (**a**) Kme3 and (**b**) TRP2, 5F-TRP2, 6F-TRP2 and 5,6diF-TRP2, computed at BLYP-D3BJ/TZ2P.

We find that the trend in the interaction energy ∆*E*_int_ originates from the electrostatic attraction Δ*V*_elstat_. This attraction is less favourable by 2.8–3.0 kcal mol^−1^ for difluorinated, and by 6.0 kcal mol^−1^ for tetrafluorinated TRP2 when compared with the TRP2 cage, a trend that we attribute to weaker cation–π interactions (Table 2). The weakening of the electrostatic potential is caused by the fact that the electronegative fluorine substituents pull electronic charge density away from the aromatic core (Fig. 5), thus reducing the quadrupole of the rings. This is clearly observed by comparison of the two extreme systems, TRP2–Kme3 and 5,6diF-TRP2–Kme3, that present the strongest effect. In the former, only one carbon in the six-membered ring acquires a net positive partial charge, whereas in the latter, four such partially positively charged carbon atoms exist (Fig. 5). The more positively charged carbon atoms in the six-membered ring reduce the quadrupole, which causes a less favourable electrostatic interaction with positively charged trimethyllysine. The effect is less pronounced for the disubstituted 5F-TRP2–Kme3 and 6F-TRP2–Kme3 systems that have two positively charged carbon atoms in the ring skeleton. Finally, the same constant behaviour as observed for ∆*E*_Pauli_ also applies to the orbital interaction term ∆*E*_oi_, with a maximum difference of 0.4 kcal mol^−1^ with fluorinated TRP2 cages (Table 2). The frontier orbitals involved in the interaction between Kme3 and TRP2 are depicted in Fig. 5 for both fragments. The incorporation of fluorine substituents onto tryptophan residues does not affect the shape of the corresponding frontier orbitals; in particular, the interaction between the donor orbitals of TRP2 and the acceptor orbitals of Kme3 is not altered. This finding is further supported by the overlap between the π orbitals of TRP2 and the acceptor orbitals of Kme3 (Supplementary Table 5), with very close values among the four different systems under analysis. The same constant behaviour is also displayed by the dispersion correction term Δ*E*_disp_, which undergoes a negligible change of 0.1 kcal mol^−1^ upon fluorination. It is noteworthy that the Δ*E*_disp_ term contributes the largest to the interaction, however, it has no effect on trends because of its relatively constant value (Table 2).

Further insight into the effect of fluorination of the aromatic cage on the interaction with Kme3 can be gained by estimating the interaction of the cationic nitrogen of Kme3 with either exclusively the five-membered ring or exclusively the six-membered ring of TRP2. We achieved this by introducing tailor-made, for this purpose, modifications into our model systems. Thus, we have constructed modifications of our TRP2–Kme3 system by just keeping one five-membered ring of one TRP unit and one six-membered ring of the other TRP unit, whereas Kme3 has been simplified to NMe_4_^+^ (the same procedure has been applied to the fluorinated systems, Supplementary Fig. 15). Next, we have calculated the energy change ∆*E* associated with the model isodesmic reaction for equilibrium between the NMe_4_^+^-6-membered ring and NMe_4_^+^-5-membered ring (Supplementary Fig. 15). ∆*E* amounts to −1.30, −1.52, −1.48 and −1.77 kcal mol^−1^ for unfluorinated, 5-monofluorinated, 6-monofluorinated, and 5,6-difluorinated systems, respectively, all computed at the same BLYP-D3BJ/TZ2P with COSMO level (Supplementary Table 6). These values reveal that the interaction of the NMe_4_^+^ cation is more favourable with the five-membered rings than with the six-membered rings by 1.3–1.8 kcal mol^−1^, with a larger difference in case of fluorinated rings. The EDA analyses performed on these systems show that the more favourable interaction of the cation with the five-membered rings is due to an accordingly more favourable electrostatic interaction between the NMe_4_^+^ and the same, in all four systems (Supplementary Table 6). This electrostatic preference goes with a shorter distance between the NMe_4_^+^ and the five-membered ring (Table 2) together with the fact that the five-membered ring is more negatively charged than the six-membered ring (336 vs. 285 mili-a.u., Supplementary Fig. 16). Furthermore, the electrostatic term ∆*V*_elstat_ is even less favourable in case of the fluorinated systems because the carbon atoms of the six-membered ring bonded to the F atoms become positively charged, thus interacting less favourably with the positively charged H atoms of NMe_4_^+^ (Supplementary Fig. 16). On the other hand, the electrostatic interaction between the NMe_4_^+^ and the five-membered ring is hardly affected by fluorination. This is in line with the fact that the five-membered rings in TRP2 are more remote from the fluorine substituents and undergo only slight changes in its atomic charges. We recall that, in the model systems discussed above, we have used, for consistency, the same distances between NMe_4_^+^ and the six- and five-membered rings as in the full TRP2 model systems. We stress however that we arrive at the same trends and conclusions if we allow for full geometrical relaxation in these further simplified model systems. Just for comparison, the equivalent isodesmic reaction energies in that case are −1.00, −1.41 and −1.79 kcal mol^−1^ for the unfluorinated, monofluorinated and difluorinated simplified model system, respectively (note that 5- and 6-substitution now lead to one and the same equilibrium geometry). Thus overall, we can conclude that the five-membered rings of TRP2 contribute more to binding to Kme3, and even more so in case of fluorinated aromatic cages.

### Water thermodynamic analysis of fluorinated aromatic cages

**Fig. 6:**
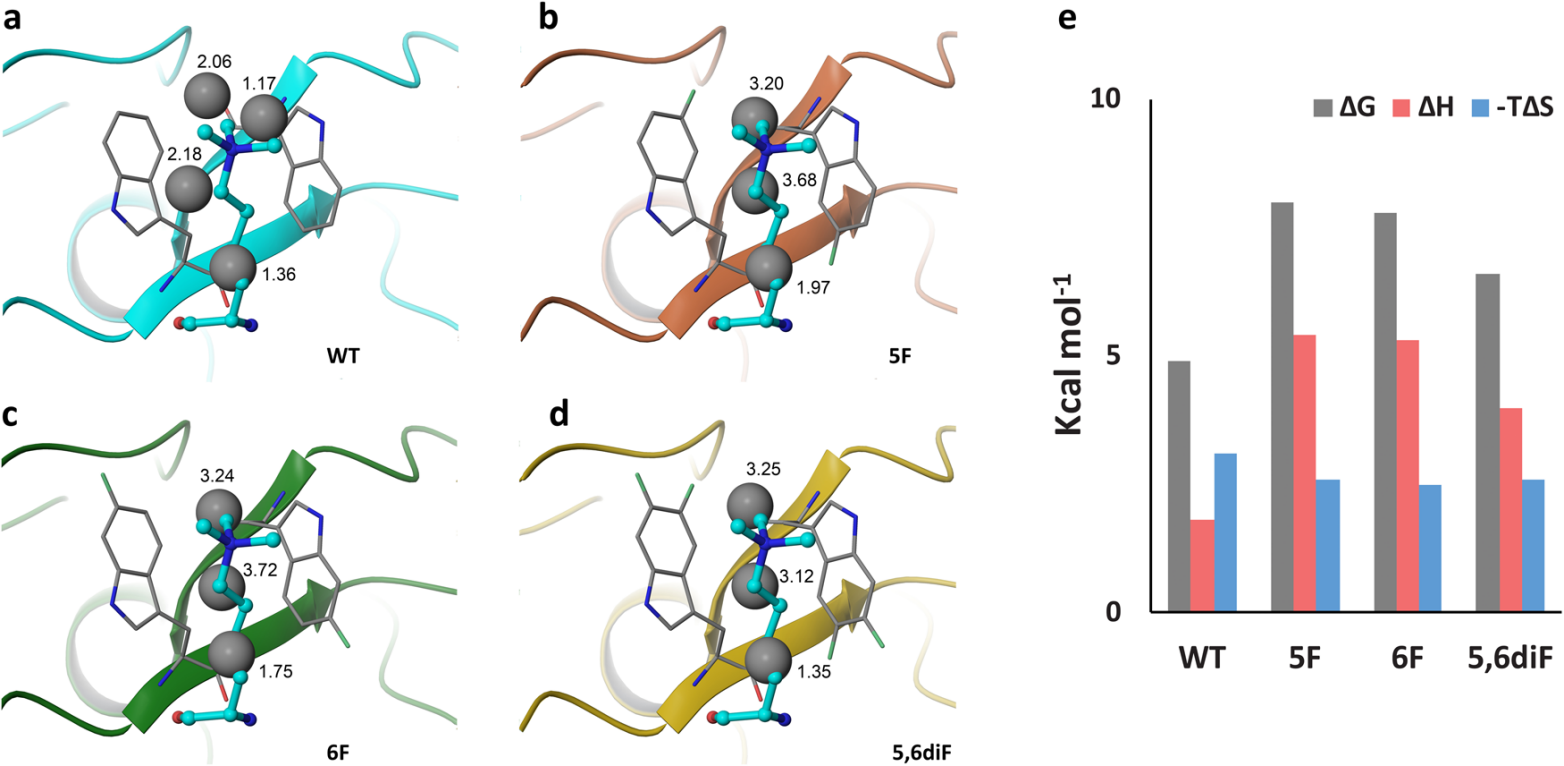
Water thermodynamic calculations for the solvation of the aromatic cage of KDM5A PHD3 fingers. **a** Wild-type KDM5A; **b** 5F-KDM5A; **c** 6F-KDM5A; **d** 5,6diF-KDM5A; **e** Thermodynamic parameters for the solvation of the aromatic cages of four KDM5A proteins used in this study.

Water thermodynamic computations, which combine MD simulations with statistical thermodynamic analysis of water molecules, provided strong evidence that desolvation of aromatic cages of trimethyllysine-binding reader proteins is energetically favourable process^16^. We conceived that fluorination of tryptophan residues that constitute the aromatic cage of the KDM5A PHD3 finger presumably leads to altered energetics of high-energy water molecules in their proximity. Therefore, water thermodynamic analyses were carried out to compute thermodynamic parameters for water molecules located in wild-type and fluorinated KDM5A (Fig. 6, Supplementary Table 7 and Supplementary Figs. 17 and 18). For wild-type KDM5A, four high-energy hydration sites were identified, whereas KDM5A PHD3 fingers that possess fluorinated tryptophan residues have three hydration sites. Despite having one water molecule fewer, fluorinated KDM5A displayed a more unfavourable free energy of solvation. The total free energy contributions from desolvation were calculated to be −4.9 kcal mol^−1^ for WT KDM5A, and −8.0, −7.8, and −6.6 kcal mol^−1^ for 5F-KDM5A, 6F-KDM5A, and 5,6diF-KDM5A, respectively (Fig. 6 and Supplementary Table 7). The increase in the free energy of solvation appears to be a result of more unfavourable enthalpy of solvation. This finding implies that fluorination of the aromatic cage results in a more favourable free energy change upon displacement of water molecules by Kme3 binding. These results support the quantum chemical analysis of the KDM5A–H3K4me3 association, as these computations predicted a compensation mechanism due to a less favourable electrostatics term and a more favourable desolvation term (Table 2). It should be noted, however, that the quantum chemically computed trend of increasingly more favourable desolvation upon 5,6-difluorination was not fully reflected by the water thermodynamic calculations, suggesting that additional energetic factors may be involved in the binding process. For example, the water thermodynamic calculations are based on a molecular mechanics force field that neglects quantum mechanical effects, whereas the quantum mechanical calculations neglect dynamic and entropic information. Despite this fact, the water thermodynamic calculations support the general conclusion that more favourable desolvation upon fluorination of the tryptophan residues constituting the KDM5A’s PHD3 aromatic cage compensates for the less favourable interactions of trimethyllysine with the weakened quadrupole of the aromatic cage.

## Discussion

Understanding the molecular origin of biomolecular recognition processes that play essential roles in human health and disease is important from a basic molecular perspective as well as from a biomedical perspective. Despite extensive examinations of non-covalent interactions in various chemical and biological systems in the past two decades^37–39^, our understanding of the underlying mechanisms that drive biomolecular recognition is partly understood at best, and among others, leads to continual difficulties in rational design of drugs that specifically bind protein targets^40,41^. The phenomenon of biomolecular recognition is further complicated by incomplete understanding of the role of water in binding processes, although recent computational efforts, in particular, have made significant advances in understanding the structure and energetics of water in protein binding pockets^42–44^. Our work highlights that cooperative experimental and computational investigations enable the examination of the recognition of trimethyllysine-containing histones by epigenetic reader KDM5A at the unprecedented level of detail. Employing a physical-organic chemistry approach allowed us to evaluate the three key contributors that dictate the readout of trimethyllysine: (i) Solute–solute interactions, i.e., cation–π interactions between the positively charged trimethyllysine and the electron-rich aromatic cage of the KDM5A PHD3 finger; (ii) Ligand desolvation, i.e., partial desolvation of trimethyllysine upon the KDM5A–Kme3 complex formation; and (iii) Protein desolvation, i.e., desolvation of the aromatic cage of KDM5A upon Kme3 binding. A strategy in which the aromatic character of tryptophan residues is perturbed by the introduction of fluorine substituents, while keeping all other parameters of the KDM5A–H3K4me3 system unaltered, eliminates the contribution from trimethyllysine desolvation in our comparative analyses (as this energetically unfavourable term is present in all systems). Our thermodynamic results (Table 1) showing that H3K4me3 interacts equally well with the electron-rich aromatic cage of wild-type KDM5A PHD3 and comparatively electron-poorer aromatic cages of fluorinated KDM5A, are markedly different to binding studies of the related protein–ligand systems; it has commonly been observed that binding of cations by fluorinated tryptophan or phenylalanine residues is governed by significantly weaker cation–π interactions^19,22,45,46^. Our MD simulations and quantum chemical analyses support these findings, by providing evidence that H3K4me3 binding to fluorinated aromatic cages of the KDM5A PHD3 finger or fluorinated TRP2 fragments is associated with an electrostatic weakening of cation–π interactions when compared with wild-type KDM5A/TRP2. Notably, the water thermodynamic calculations on the PHD3 finger of KDM5A that possesses tryptophan or its fluorinated counterparts reveal that the energetics of water molecules that occupy aromatic cages is altered upon fluorination of the tryptophan residues. While 3–4 high-energy water molecules are present inside all aromatic cages, the free energy of solvation is more unfavourable in aromatic cages comprised of fluorinated tryptophan residues; these results are line with an increased hydrophobicity of fluorinated benzene relative to benzene^47^. Collectively, our thermodynamic binding studies and computational analyses reveal that the association between the positively charged trimethyllysine and F-substituted tryptophan residues that constitute the aromatic cage of the PHD3 domain of KDM5A is maintained by weaker cation–π interactions (when compared with the wild-type aromatic cage) that are compensated by energetically more favourable desolvation of aromatic cages (when compared with the wild-type aromatic cage) upon trimethyllysine binding. More detailed examinations of biomolecular recognition of histones will greatly contribute to our basic understanding of the histone code^48^, which postulates that the molecular landscape of posttranslational modifications on histone proteins is tightly associated with interactions with chromatin-associated proteins, thus altering the chromatin structure and function.

This work demonstrates that a holistic physical-organic chemistry approach, based on synergistic experimental and computational tools, enables a more advanced understanding of biomolecular recognition of trimethyllysine-containing histones by epigenetic reader proteins. It is envisioned that compelling physical-organic chemistry approaches, which collectively examine non-covalent interactions and desolvation effects, along with modern chemical biology approaches^49–52^ will importantly contribute to a better understanding of underlying molecular mechanisms that govern the specific recognition of other types of posttranslational modifications found on histones and other proteins.

## Methods

### Synthesis of 5,6-difluorotryptophan

Supplementary Fig. 19 shows the schematic presentation of the synthetic protocol for the preparation of 5,6-difluorotryptophan. A suspension of 5,6-difluoroindole (501 mg, 3.27 mmol, 1 equiv.) and L-serine (688 mg, 6.54 mmol, 2 equiv.) in AcOH and Ac_2_O (18 mL, 5:1) was heated to 70 °C under Ar atmosphere in microwave vial. After 16 hours of stirring the solvent was coevaporated with toluene. Crude brown oil was purified by column chromatography (MeOH in CH_2_Cl_2_ (0–5%) and with AcOH (0.1%)), affording *N*-acetyl-5,6-difluorotryptophan (680 mg, 2.41 mmol, 74%) as a yellowish oil. ^1^H NMR (400 MHz, CD_3_OD) δ: 7.36–7.29 (m, 1 H), 7.17–7.11 (m, 1 H), 7.11 (d, *J* = 4.0 Hz, 1 H), 4.65 (dt, *J* = 8.0, 5.0 Hz, 1 H), 3.23 (ddd, *J* = 15.0, 8.0, 0.5 Hz, 1 H), 3.08 (ddd, *J* = 14.8, 8.0, 0.5 Hz, 1 H), 1.89 (s, 3 H). ^13^C NMR (101 MHz, CD_3_OD) δ: 173.5, 171.7, 131.5 (d, *J*_C-F_ = 21.5 Hz), 124.7 (d, J = 3.5 Hz), 122.8 (d, *J*_C-F_ = 7.5 Hz), 110.2 (d, *J*_C-F_ = 4.5 Hz), 104.3 (d, *J* = 18.5 Hz), 98.4 (d, *J*_C-F_ = 21.5 Hz), 53.2, 20.9. ESI-MS calcd for C_13_H_13_F_2_O_3_N_2_ [M + 1]^+^ 283.0894, found 283.0888. A solution of *N*-acetyl-5,6-difluorotryptophan (485 mg, 1.71 mmol) was dissolved in aqueous NaOH (25 mL, 4 M) and heated at 100 °C for 16 h. The reaction mixture was then cooled and acidified to pH 1. Solvent was evaporated and the crude product was purified by preparative HPLC, affording racemic 5,6-difluorotryptophan as a TFA salt (140 mg, 0.39 mmol, 23%) as a yellowish oil. ^1^H NMR (400 MHz, CD_3_OD) δ: 7.44–7.36 (m, 1 H), 7.26–7.17 (m, 2 H), 4.21 (dd, *J* = 7.0, 5.0 Hz, 1 H), 3.39 (ddd, *J* = 15.5, 5.0, 0.5 Hz, 1 H), 3.30 (ddd, *J* = 15.5, 5.0, 0.5 Hz, 1 H). ^13^C NMR (101 MHz, CD_3_OD) δ: 170.2, 148.1 (dd, *J*_C-F_ = 168.0, 15.5 Hz), 145.7 (dd, *J*_C-F_ = 165.0, 15.5 Hz), 131.9 (d, *J*_C-F_ = 10.5 Hz), 126.0 (d, *J*_C-F_ = 3.5 Hz), 122.3 (d, *J*_C-F_ = 7.0 Hz), 106.9 (d, *J*_C-F_ = 5.5 Hz), 104.3 (d, *J*_C-F_ = 19.5 Hz), 98.8 (d, *J*_C-F_ = 21.5 Hz), 52.9, 25.9. ^19^F NMR (377 MHz, MeOD) δ −78.2 (s, 3 F), −148.3 (m, 1 F), −151.6 (m, 1 F). ESI-MS calcd for C_11_H_11_F_2_O_2_N_2_ [M + 1]^+^ 241.0789, found 241.0797.

### Auxotrophic production of KDM5A

The wild-type KDM5A PHD3 finger (Homo sapiens, uniport ID: P29375, residues 1598–1663) fused to GST was expressed in Rosetta BL21 (DE3)pLysS *E. coli* containing the KDM5A-GST construct in TB medium supplemented with the appropriate antibiotics. At OD_600_ ~0.6, expression was induced with 0.4 mM IPTG and 0.1 mM ZnCl_2_ (final concentration) and cultured overnight at 16 °C. Cells were then harvested, lysed and purified using GST affinity. The GST tag was cleaved off with TEV-protease under reducing conditions (10 mM dithiothreitol), and the KDM5A PHD3 finger was subsequently purified by size exclusion chromatography on a Superdex 75 column using 20 mM TRIS-HCl pH 7.5, 50 mM NaCl, 1 mM DTT as running buffer. Protein concentration was measured spectrophotometrically using a Denovix DS-11 spectrophotometer and protein masses were confirmed by ESI-MS analyses. Wild-type and fluorinated PHD3 finger of KDM5A-GST (Homo sapiens, uniport ID: P29375, residues 1598–1663) expressed in the auxotrophic *E. coli* (Migula) Castellani and Chalmers strain were cultured in either New Minimal Medium (NMM) or in Unnatural amino acid New Minimal Medium (UNMM) supplemented with appropriate antibiotics, respectively. NMM was prepared as described by Budisa and coworkers^27,28^. In brief, NMM contained 100 mM K_2_HPO_4_, 55 mM KH_2_PO_4_, 20 mM D-glucose, 8.5 mM NaCl, 7.5 mM (NH_4_)_2_SO_4_, 1 mM MgSO_4_, 10 mg l^−1^ biotin, 10 mg l^−1^ Thiamine-HCl, 1 mg l^−1^ CaCl_2_ and FeCl_3_, 1 μg l^−1^ CuSO_4_, MnCl_2_, ZnCl_2_, NaMoO_4_ and 50 mg l^−1^ of each individual amino acid. UNMM was prepared similarly except that tryptophan was substituted by the desired fluorinated tryptophan analogue, at a final concentration of 25 mg l^−1^. *E. coli* (Migula) Castellani and Chalmers containing the wild-type KMD5A construct was cultured in NMM at 37 °C. At OD_600_ ~0.6, the NMM medium was refreshed by harvesting the cells, after which they were resuspended in fresh NMM. Expression was then induced with 0.1 mM IPTG and 0.1 mM ZnCl_2_ (final concentrations). The cells were subsequently cultured for 3 h at 37 °C, after which the culture was harvested, lysed and purified as described above. Fluorinated tryptophan analogues were introduced into KDM5A as follows: *E. coli* (Migula) Castellani and Chalmers containing the KDM5A construct was initially cultured in NMM at 37 °C. At OD_600_ ~0.6, the cells were harvested and subsequently washed three times with 0.9% NaCl at room temperature. Following the washing steps, the cells were resuspended in fresh UNMM. Expression was then induced with 1.0 mM IPTG and 0.1 mM ZnCl_2_ (final concentrations). The cells were subsequently cultured for 3 h at 37 °C after which the culture was harvested, lysed and purified as described above.

### Circular dichroism

CD experiments were carried out at a protein concentration of 0.1 mg ml^−1^ in 10 mM phosphate buffer, pH 7.5, on a J-815 circular dichroism spectropolarimeter. The samples were measured over a range of 180–260 nm with normal sensitivity and a bandwidth of 1 nm. Scanning was performed at 50 nm per minute, a data integration time (D.I.T.) of 0.5 s and a data pitch of 0.5 nm. The spectra for each protein are a result of 10 accumulations.

### Differential scanning fluorimetry

Protein melt curves were obtained as described by Reinhard et al. using a StepOne-Plus Real-Time PCR system (Applied Biosystems) and MicroAmp fast optical 96-well reaction plates (Applied Biosystems)^53^. SYPRO-Orange protein gel stain (Invitrogen) was used as a reporter dye, emitting fluorescence in the FAM channel. Total reaction volume was 25 μl:20 μl buffer (25 mM TRIS-HCl pH 7.5, 50 mM NaCl, 1 mM DTT), 2.5 μl of 25 μM protein and 2.5 μl SYPRO-Orange dye (diluted 1:100 in ddH_2_O). Melt curve data were obtained in triplicate, in a temperature range of 20–95 °C at a stepwise temperature increment of 1 °C min^−1^. Obtained data were analyzed using DSF Analysis v3.0.2 software, designed by Niesen et al. (available via ftp://ftp.sgc.ox.ac.uk/pub/biophysics/)^54^.

### Isothermal titration calorimetry

The same batch of H3K4me3 histone peptide (ARTKme3QTARKS, 380 μM) was titrated to all KDM5A PHD3 fingers (28 μM). Due to lower expression of the auxotrophic WT-KDM5A, H3K4me3 (190 μM) and AUX WT-KDM5A (21 μM) were used. The buffer used for ITC experiments was the same as the elution buffer used for SEC; 20 mM TRIS-HCl pH 7.5, 50 mM NaCl, 1 mM DTT. Each ITC titration consisted of 19 injections. ITC experiments were performed on the fully automated Microcal Auto-iTC200 (GE Healthcare Life Sciences, USA). Heats of dilution for histone peptides were determined in control experiments, and were subtracted from the titration binding data before curve fitting. Curve fitting was performed by Origin 6.0 (Microcal Inc., USA) using one set of sites binding model. With the exception of the auxotrophic WT-KDM5A–H3K4me3 (replicate), 7–9 independent ITC experiments were carried out for other four reader–histone systems.

### ^19^F NMR spectroscopy

Measurements were obtained on a Bruker AVANCE III 400 MHz system equipped with a BBFO probe capable of ^19^F nucleus detection with ^1^H decoupling. Samples were prepared using 5 mm Shigemi tubes matched to D_2_O to minimize solvent volume required. ^19^F NMR experiments were performed 10 mM H_2_KPO_4_ pH 7.5, at a concentration of 450 μM of 5F-KDM5A/6F-KDM5A and 1 mM of H3K4me3 peptide (ARTKme3QTARKS). All measurements were performed at 288 K. After samples were inserted into the magnet, the sample was shimmed using the lock nucleus in D_2_O and a ^1^H spectrum was acquired to assess the quality of the shims. The probe was then manually tuned and matched to ^19^F to optimize ^19^F detection. A 15 μs @ 23 Watts 90-degree pulse was used. ^19^F{^1^H} spectra were then acquired with the following parameters: NS = 1.5 k–28 k, d1 = 1, aq = 1.09 s, sw = 20.1 ppm and o1p near −120 ppm. ^19^F NMR spectra were externally referenced to CFCl_3_ using the frequency of residual solvent signal in the ^1^H spectrum and the ratio between the ^1^H and ^19^F gyromagnetic ratios.

### MD simulations

Four MD simulations were carried out for 100 ns each using the Amberff12SB force field. A PDB structure for the model representing KDM5A PHD3 (PDB: 2KGI) was used as a template for building the readerKme3 systems. KDM5A residues Trp18 and Trp28 were manually modified to generate the 5F-Trp18/5F-Trp28, 6F-Trp18/6F-Trp28, and 5,6diF-Trp18/5,6diF-Trp28 complexes. Hydrogen atom addition was performed with LEaP. Systems were solvated in a 10 Å truncated octahedral box of TIP3P^32^ water and neutralized explicitly with either sodium or chloride counterions. Non-bonding parameters of Zn(II) previously established from studies of KDM4A^35^ were employed. Atomic partial charges for 5F-Trp, 6F-Trp, and 5,6diF-Trp correspond to the Restrained Electrostatic Potential (RESP)^55^ charges, as shown in Supplementary Tables 8–10. Parameters for Kme3 were taken from previous work^29^. The final systems were minimized for 1000 cycles of steepest-descent minimization followed by 1000 cycles of conjugate-gradient minimization to remove close van der Waals contacts using the sander program in AMBER12. Equilibration was achieved using PMEMD to heat the systems to 310 K followed by independent MD simulations performed with a periodic boundary condition at a constant pressure of 1 atm with isotropic molecule-based scaling at a time step of 2.0 fs. All simulations used a dielectric constant of 1.0, Particle Mesh Ewald summation^56^ to calculate long-range electrostatic interactions and bond-length constraints applied to all bonds to H atoms. Trajectories were saved at 20 ps intervals and visualized using VMD^57^. Electrostatic energies between the terminal modified Kme3 side chain (e)-N atom and the π-system of surrounding aromatic cages were calculated with the NAMDEnergy Plugin^57^. The π-system was defined for tryptophan and fluorinated tryptophans as the side chain indole ring (non-H) atoms. Energy values were measured every 20 ps and averaged over 100 ns.

### Quantum chemical analyses

All calculations were carried out with the Amsterdam Density Functional (ADF) program using dispersion-corrected density functional theory at the BLYP-D3BJ/TZ2P level of theory^36,58^. The effect of solvation was simulated by means of the Conductor-like Screening Model (COSMO) of solvation as implemented in ADF. The approach has been benchmarked against highly correlated post-Hartree-Fock methods and experimental data and was found to work reliably^59–62^.

The bonding mechanism in our model complexes have been further analysed using quantitative (Kohn-Sham) molecular orbital (MO) theory in combination with an energy decomposition analysis (EDA)^63,64^. The bond energy in aqueous solution ∆*E*(aq) consists of two major components, namely, the strain energy ∆*E*(aq)_strain_ associated with deforming the Kme3 and the reader from their own equilibrium structure to the geometry they adopt in the complex, plus the interaction energy ∆*E*(aq)_int_ between these deformed solutes in the complex (Eq. 1):

1 $$\Delta {\it{E}}\left( {{\mathrm{aq}}} \right) = \Delta {\it{E}}\left( {{\mathrm{aq}}} \right)_{{\mathrm{strain}}} \,+\, \Delta {\it{E}}\left( {{\mathrm{aq}}} \right)_{{\mathrm{int}}}$$

To arrive at an understanding of the importance of desolvation phenomena during the complexation process, we separate the solute–solute interaction ∆*E*(aq)_int_ into the effect caused by the change in solvation ∆*E*(desolv) and the remaining intrinsic interaction ∆*E*_int_ between the unsolvated fragments in vacuum ∆*E*_int_:

2 $$\Delta {\it{E}}\left( {{\mathrm{aq}}} \right)_{{\mathrm{int}}} = \Delta {\it{E}}\left( {{\mathrm{desolv}}} \right)_{{\mathrm{int}}}\, +\, \Delta {\it{E}}_{{\mathrm{int}}}$$

In the EDA, the intrinsic interaction energy Δ*E*_int_ can be further decomposed as shown in Eq. 3:

3 $$\Delta {\it{E}}_{{\mathrm{int}}} = \Delta {\it{V}}_{{\mathrm{elstat}}} + \Delta {\it{E}}_{{\mathrm{Pauli}}} + \Delta {\it{E}}_{{\mathrm{oi}}} + \Delta {\it{E}}_{{\mathrm{disp}}}$$

Here, ∆*V*_elstat_ corresponds to the classical electrostatic interaction between the unperturbed charge distributions of the deformed fragments which is usually attractive. The Pauli repulsion ∆*E*_Pauli_ comprises the destabilizing interactions between occupied orbitals and is responsible for the steric repulsions. The orbital interaction ∆*E*_oi_ accounts for charge transfer (donor–acceptor interactions between occupied orbitals on one moiety with unoccupied orbitals of the other, including the HOMO–LUMO interactions) and polarization (empty/occupied orbital mixing on one fragment due to the presence of another fragment). Finally, the ∆*E*_disp_ term accounts for the dispersion interactions based on Grimme’s DFT-D3BJ correction. Furthermore, the charge distribution has been analysed using the Voronoi deformation density (VDD) method^65^.

### Water thermodynamic calculations

Water thermodynamic calculations were performed with the program WaterMap, as described in previous reports^66,67^. All calculations were run in with default settings. In brief, a 2 ns molecular dynamic (MD) simulation of the KDM5A PHD3 finger with the histone peptide removed, was performed using the Desmond molecular dynamic engine with the OPLS2.1 force field^43^. Protein atoms were constrained throughout the simulation. Water molecules from the simulation were then clustered into hydration sites for thermodynamic analysis. Enthalpy values for each hydration site were obtained by computing the average non-bonded interaction for each water molecule in the cluster over the course of the MD simulation. Entropy values were calculated using a numerical integration of a local expansion of the entropy in terms of spatial and orientational correlation functions^68,69^. The contribution of water-free energy to the binding free energy of the peptide was approximated by the sum of the free energies of hydration sites displaced by the ligand upon binding.

### Reporting summary

Further information on research design is available in the Nature Research Reporting Summary linked to this article.

## Supplementary information

Supplementary Information

Reporting Summary

## Data Availability

The authors declare that the main data supporting the findings of this study are available within the paper and its Supplementary Information file. Other relevant data are available from the corresponding author upon reasonable request.
