# Supplementary material for: Mechanism of biomolecular recognition of trimethyllysine by the fluorinated aromatic cage of KDM5A PHD3 finger
Source: Commun Chem. 2020 Jun 1;3:69. doi: 10.1038/s42004-020-0313-2 (PMC9814790; doi:10.1038/s42004-020-0313-2)
Supplement: Supplementary file 1 — Supplementary Information [file 42004_2020_313_MOESM1_ESM.pdf]

Supplementary Information

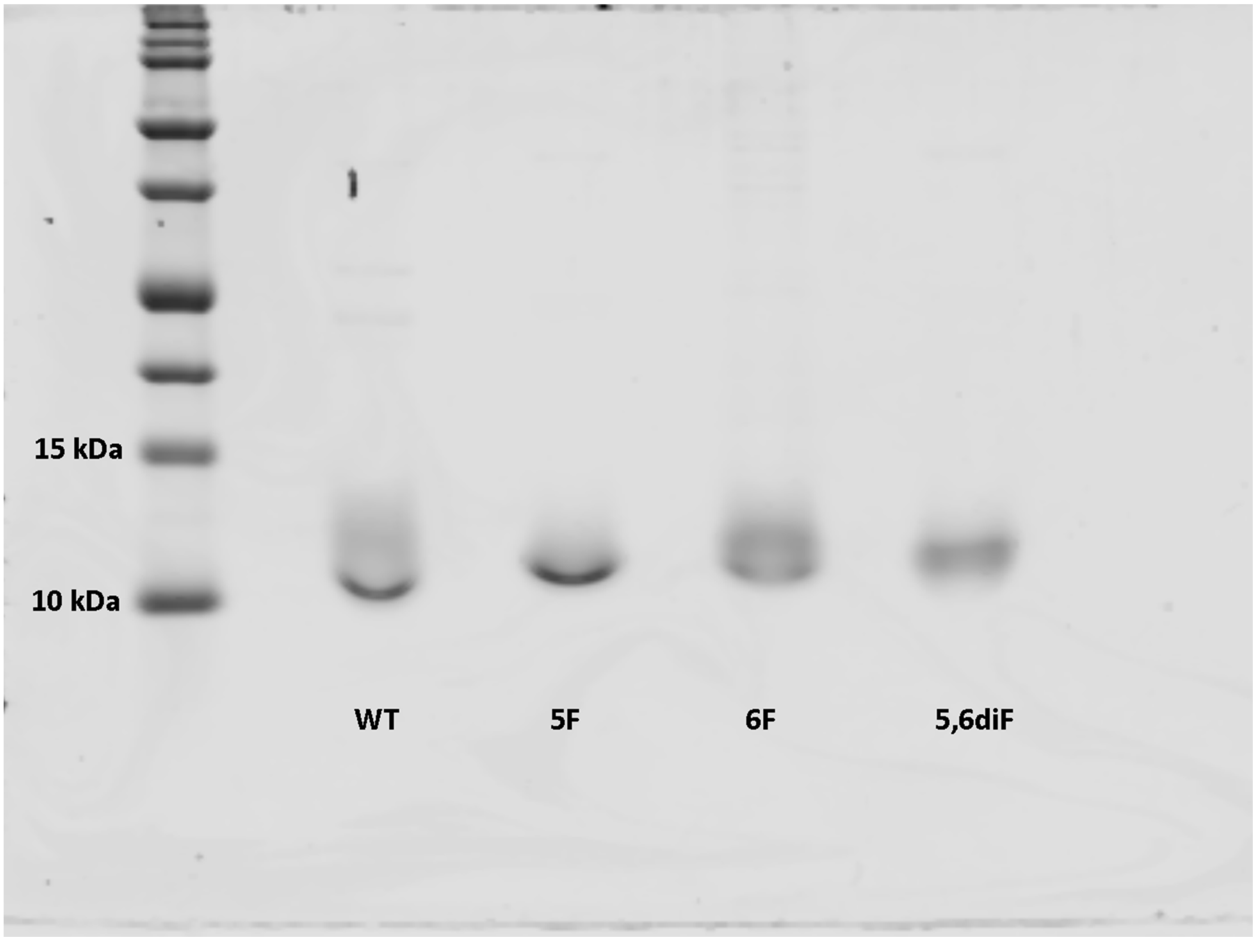

Supplementary Figure 1. Full 15% Tris-Tricine SDS-PAGE gel showing purified, untagged KDM5A PHD3 fingers.

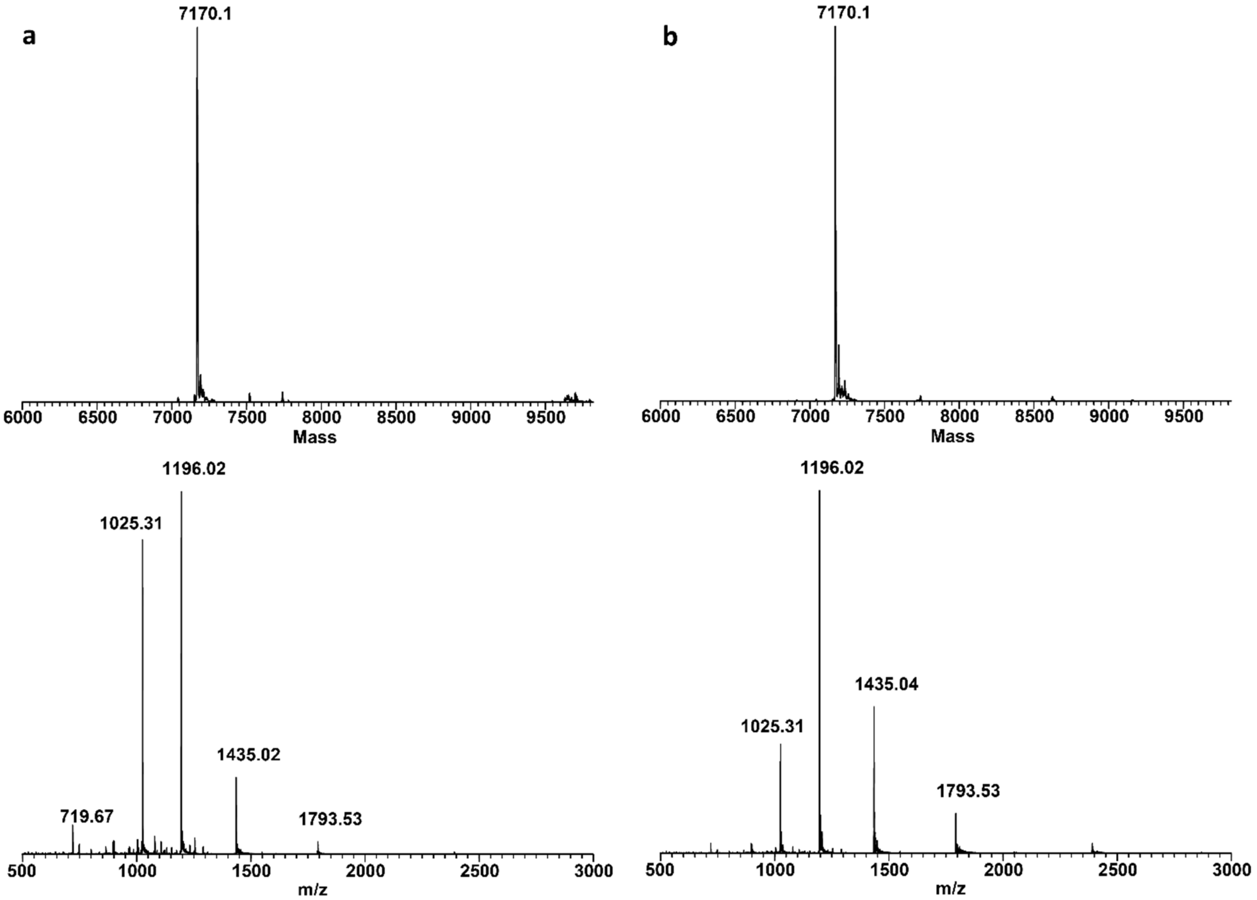

Supplementary Figure 2. ESI-MS data of the wild-type PHD3 finger of KDM5A. KDM5A reader domain expressed in (**a**) *E. coli* Rosetta BL21 (DE3)pLysS and (**b**) the auxotrophic *E. coli* Castellani and Chalmers strain, respectively.

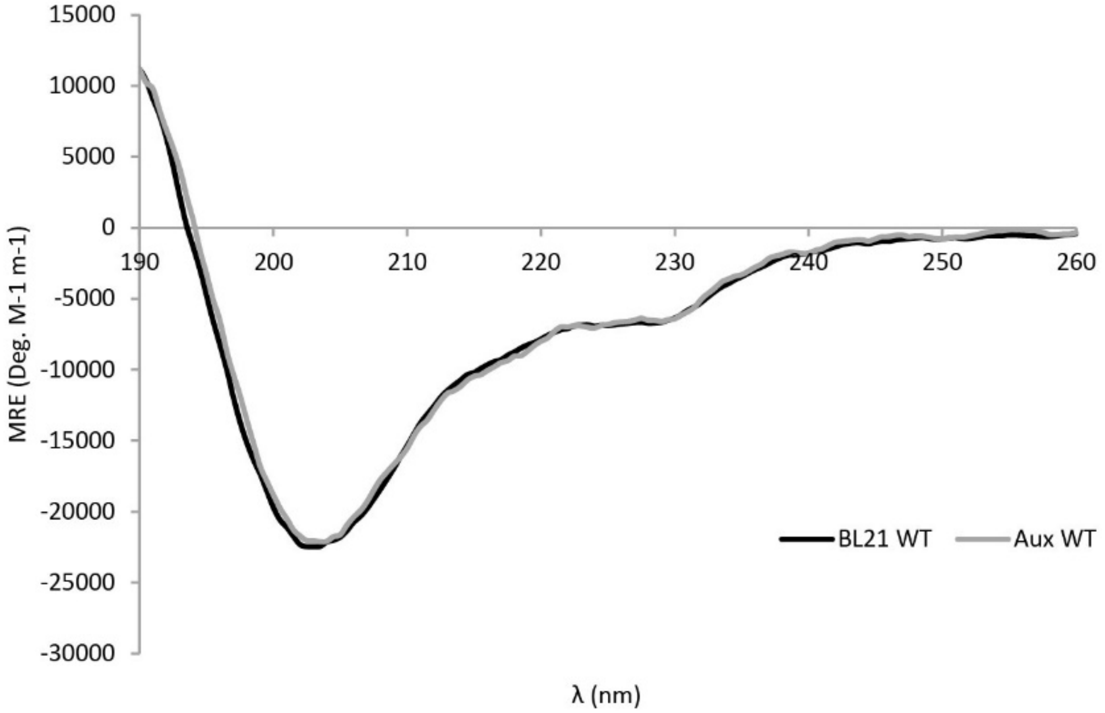

Supplementary Figure 3. CD spectra of the wild-type PHD3 finger of KDM5A. Wild-type KDM5A reader domain expressed in *E. coli* Rosetta BL21 (DE3)pLysS is displayed in black and the auxotrophic *E. coli* Castellani and Chalmers in grey, respectively.

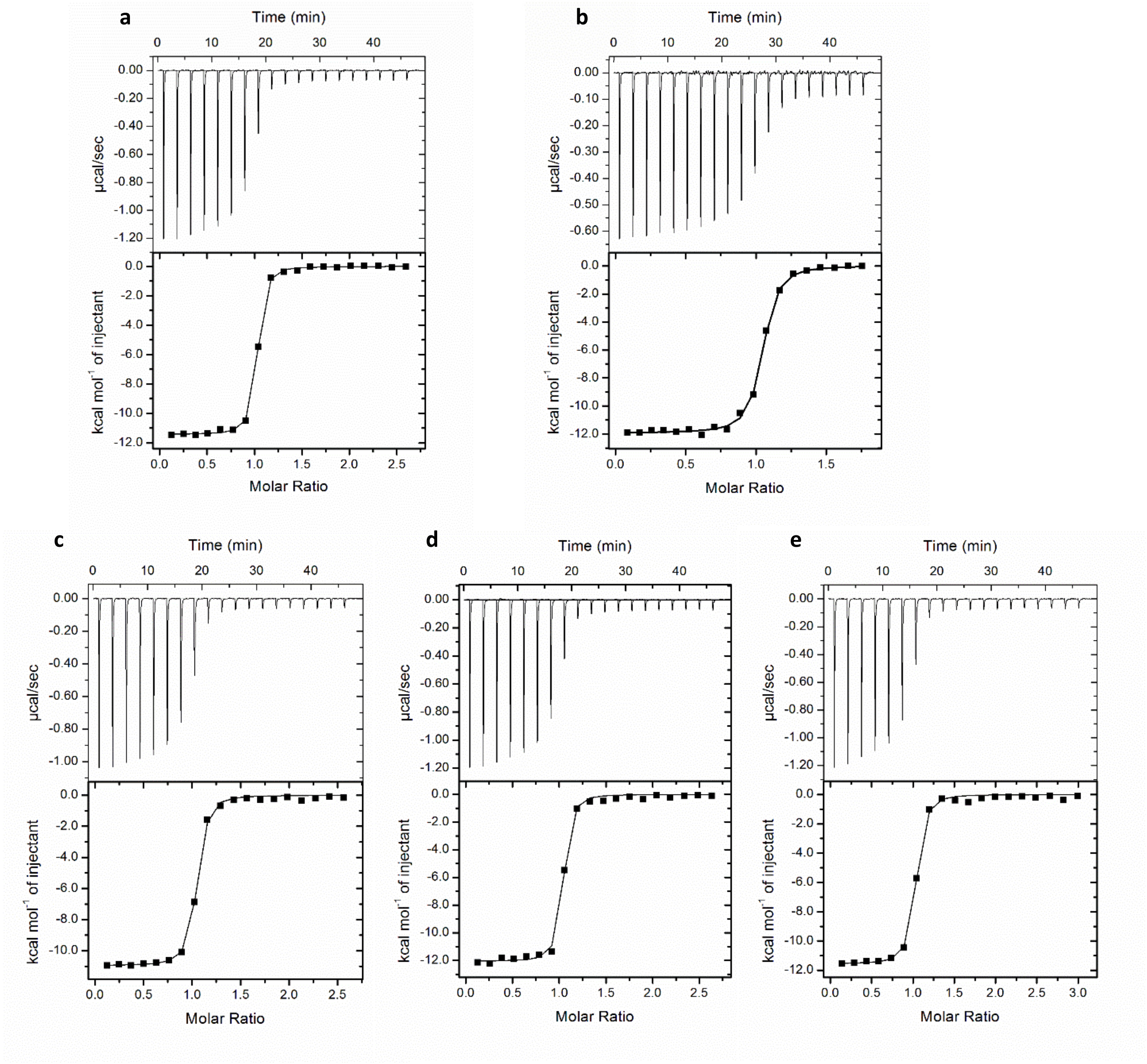

Supplementary Figure 4. ITC data for binding of H3K4me3 to wild-type and fluorinated PHD3 fingers of KDM5A. (**a**) BL21 WT-KDM5A–H3K4me3; (**b**) AUX WT-KDM5A–H3K4me3; (**c**) 5F-KDM5A–H3K4me3; (**d**) 6F-KDM5A–H3K4me3; (**e**) 5,6diF-KDM5A–H3K4me3.

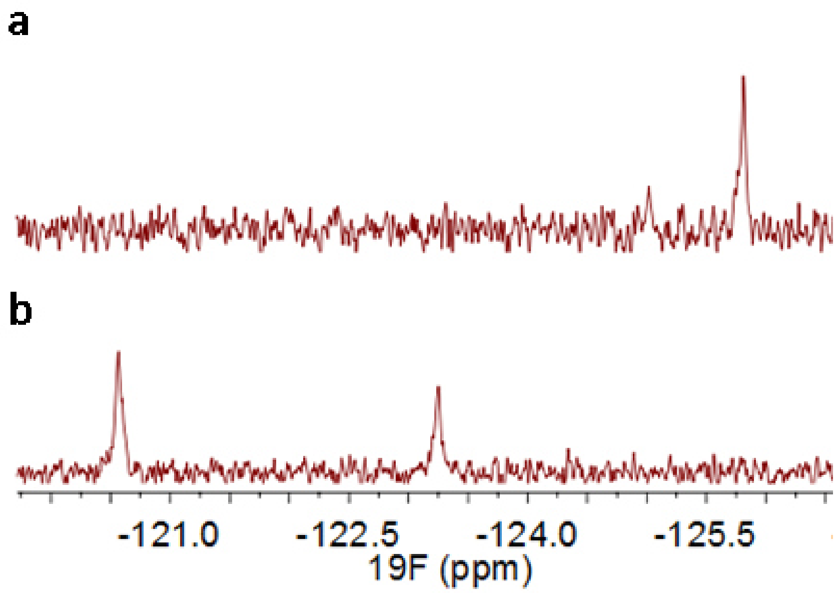

Supplementary Figure 5. ^{19}F NMR spectra of (**a**) 5F-KDM5A and (**b**) 5F-KDM5A in the presence of the H3K4me3 peptide.

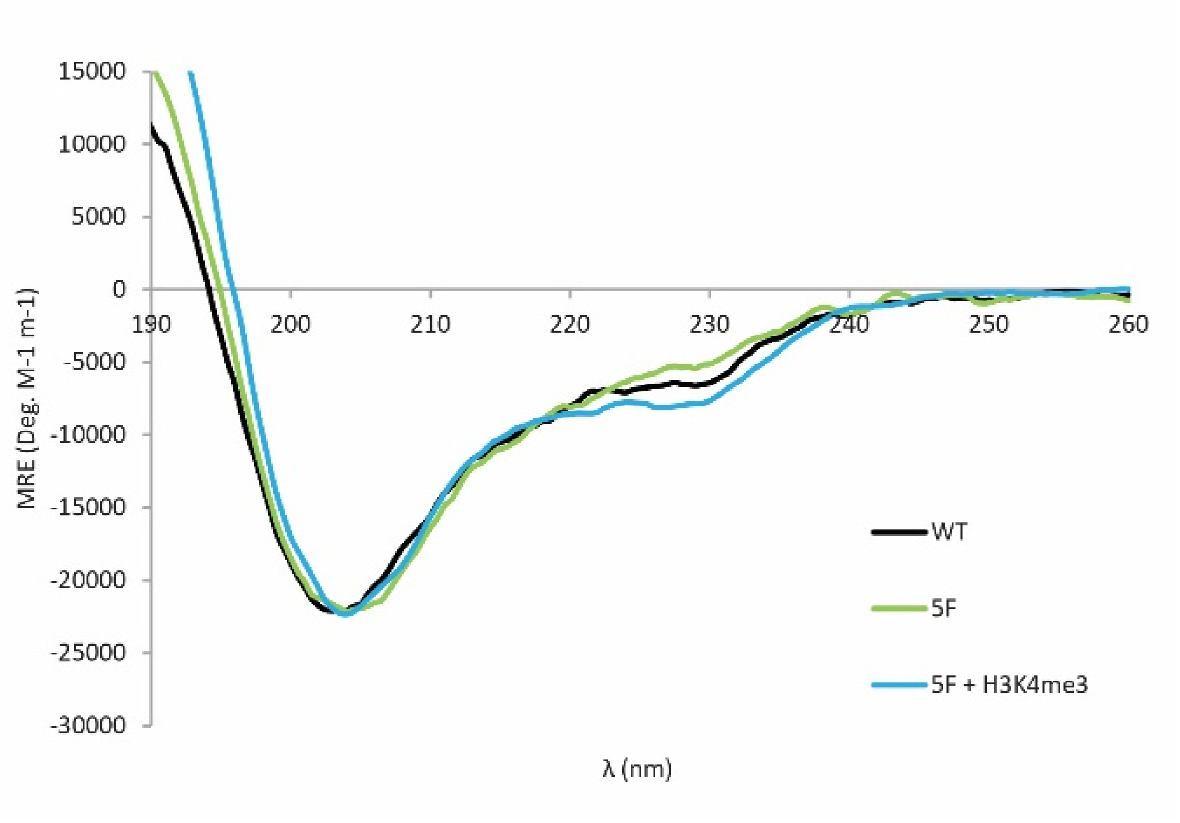

Supplementary Figure 6. CD spectra of 5F-KDM5A in the presence and absence of the H3K4me3 peptide (ARTKme3QTARKS). CD spectra are taken for the purified, untagged PHD3 domain of 5F-KDM5A over a range of 190–260 nm in 10 mM NaH_2PO_4 buffer. Black: Wild-type protein expressed in *E. coli* Rosetta BL21 (DE3) pLysS; green: 5F-KDM5A; blue: 5F-KDM5A + H3K4me3 peptide (ARTKme3QTARKS).

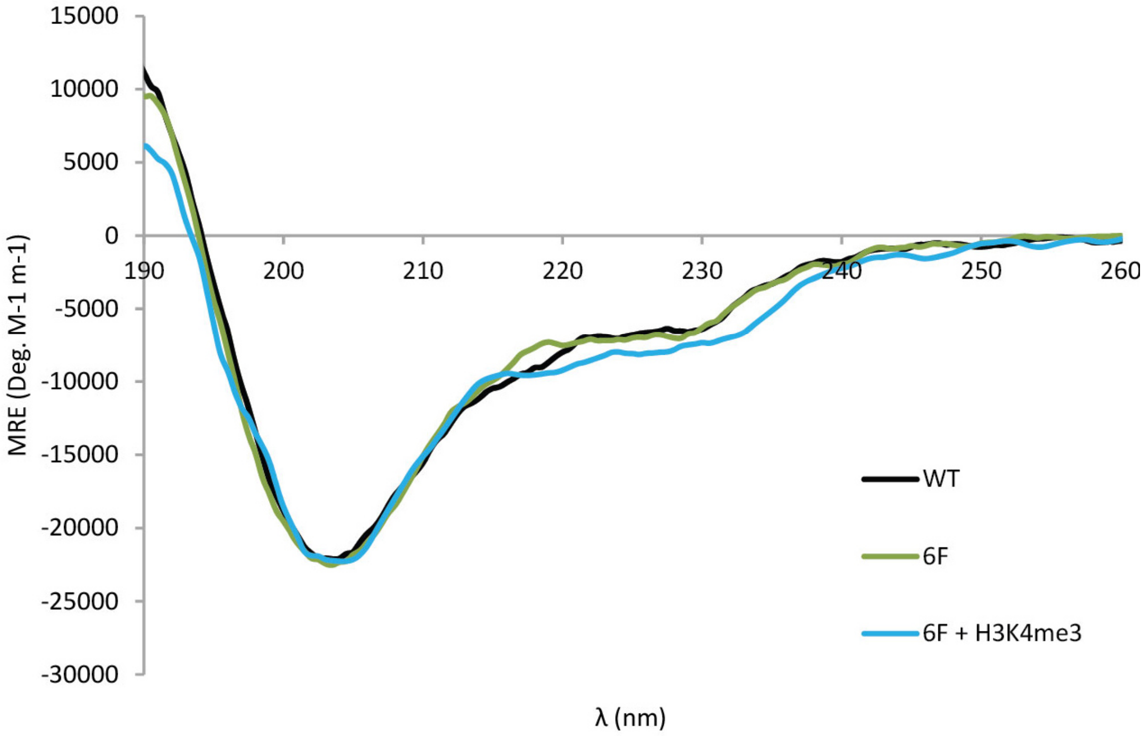

Supplementary Figure 7. CD spectra of 6F-KDM5A in the presence and absence of the H3K4me3 peptide (ARTKme3QTARKS). CD spectra are taken for the purified, untagged PHD3 domain of 6F-KDM5A over a range of 190–260 nm in 10 mM NaH_2PO_4 buffer. Black: Wild-type protein expressed in *E. coli* Rosetta BL21 (DE3) pLysS; green: 6F-KDM5A; blue: 6F-KDM5A + H3K4me3 peptide (ARTKme3QTARKS).

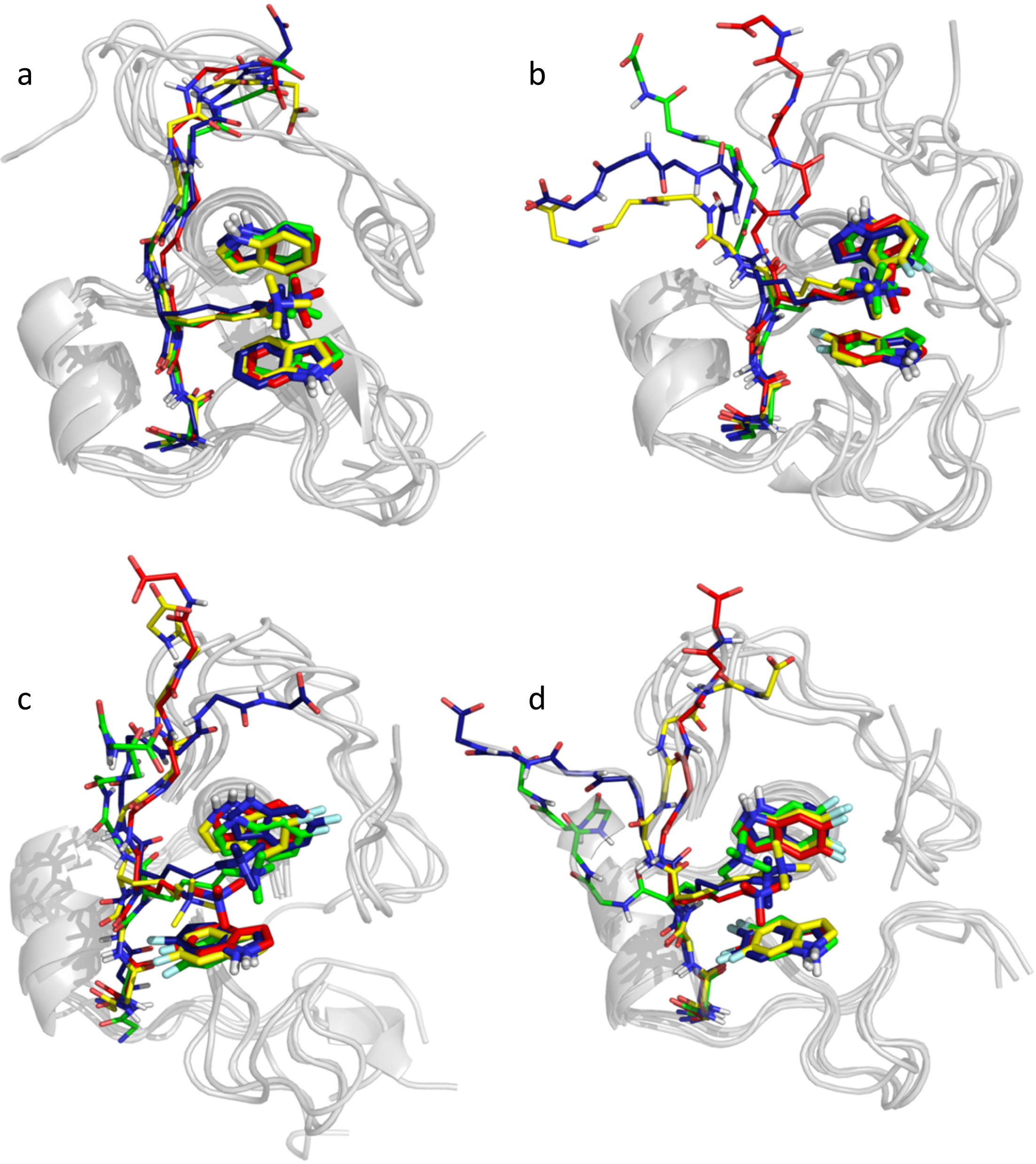

Supplementary Figure 8. Visualization of molecular dynamics simulation for (**a**) wild-type PHD3 KDM5A finger bound to H3K4me3 (PDB: 2KGI), and mutated KDM5A containing (**b**) 5F-Trp18/5F-Trp28, (**c**) 6F-Trp18/6F-Trp28, and (**d**) 5,6diF-Trp18/5,6diF-Trp28. Reader protein (grey, cartoon) binds to the H3 chain (RGB, lines) where the terminal trimethylamine of H3K4me3 occupies the Trp18-Trp28 aromatic cage (RGB, sticks). Red, yellow, green, and blue represent structures at 0 ns, 10 ns, 50 ns, and 100 ns, respectively.

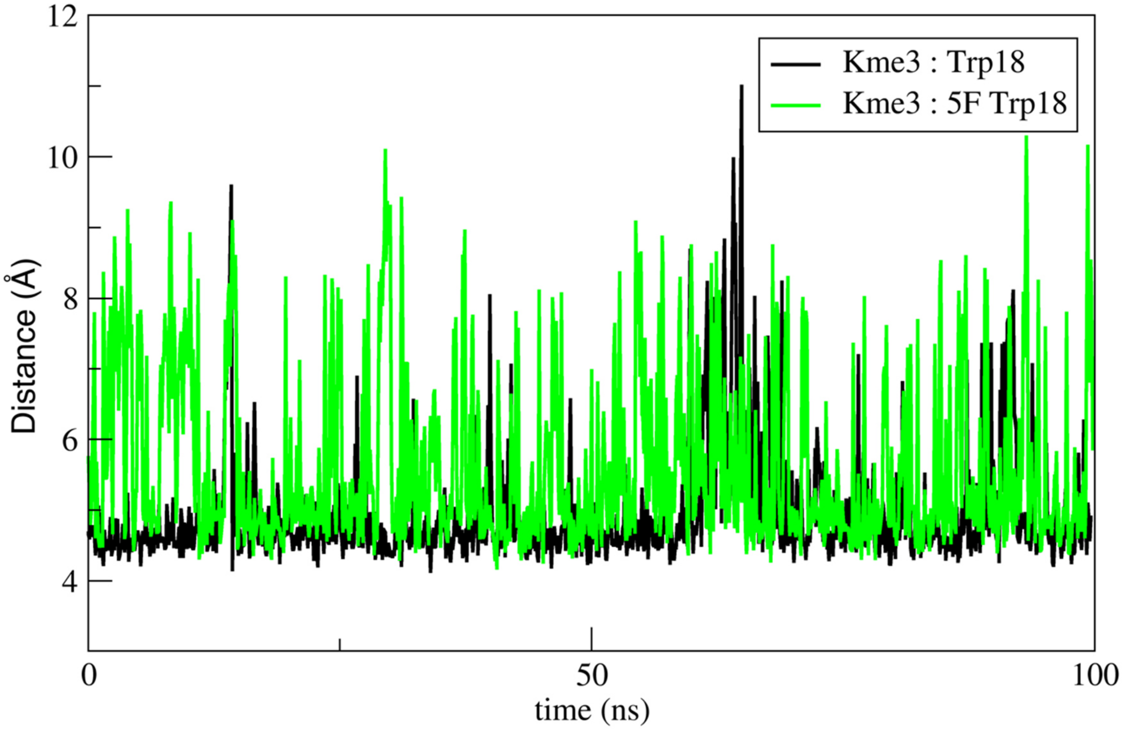

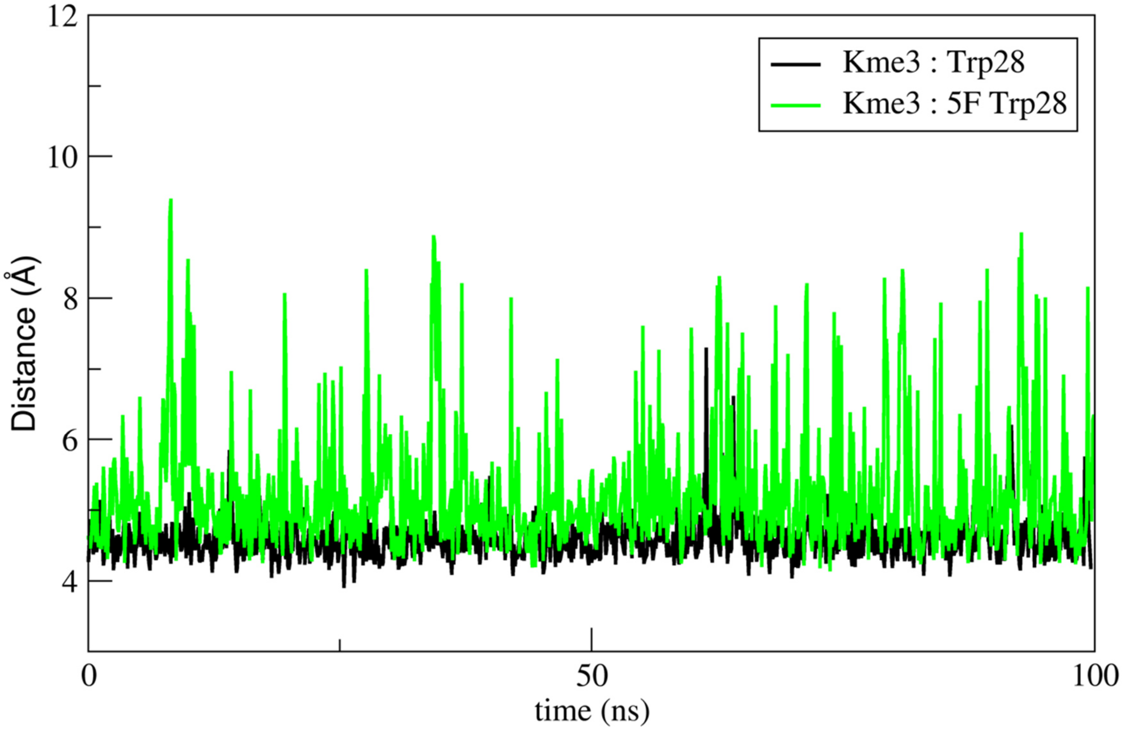

Supplementary Figure 9. Distance vs. time plots of N^+ atom of Kme3 to Trp18, Trp28, 5F-Trp18, and 5F-Trp28 side chain center of mass over 100 ns.

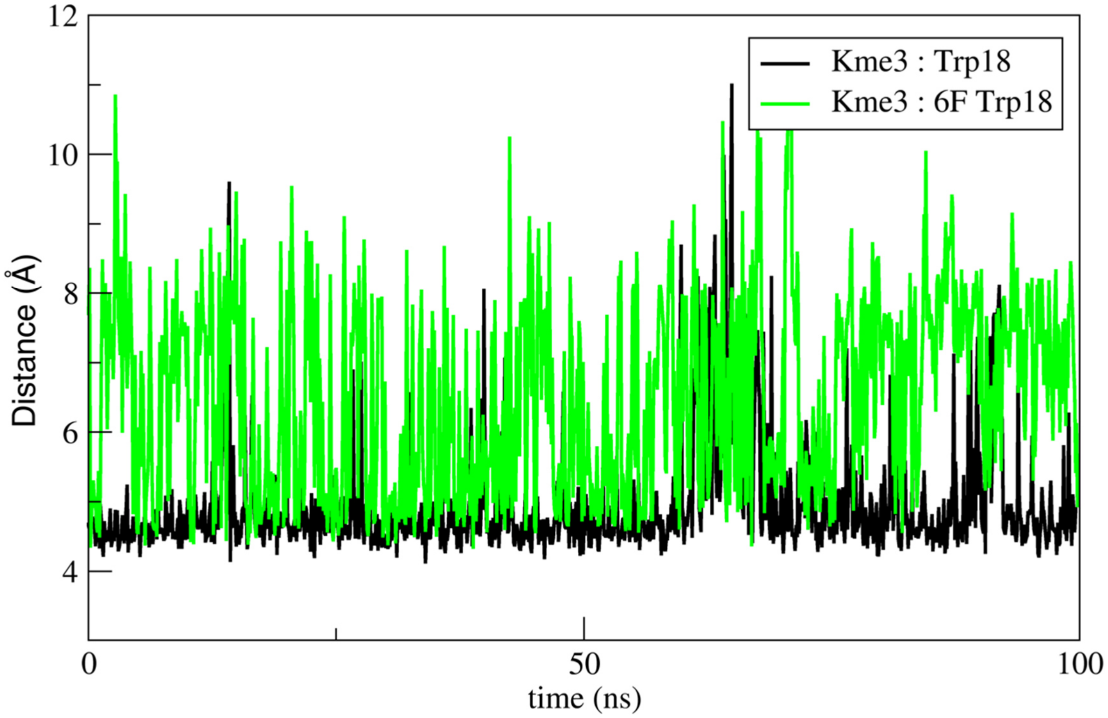

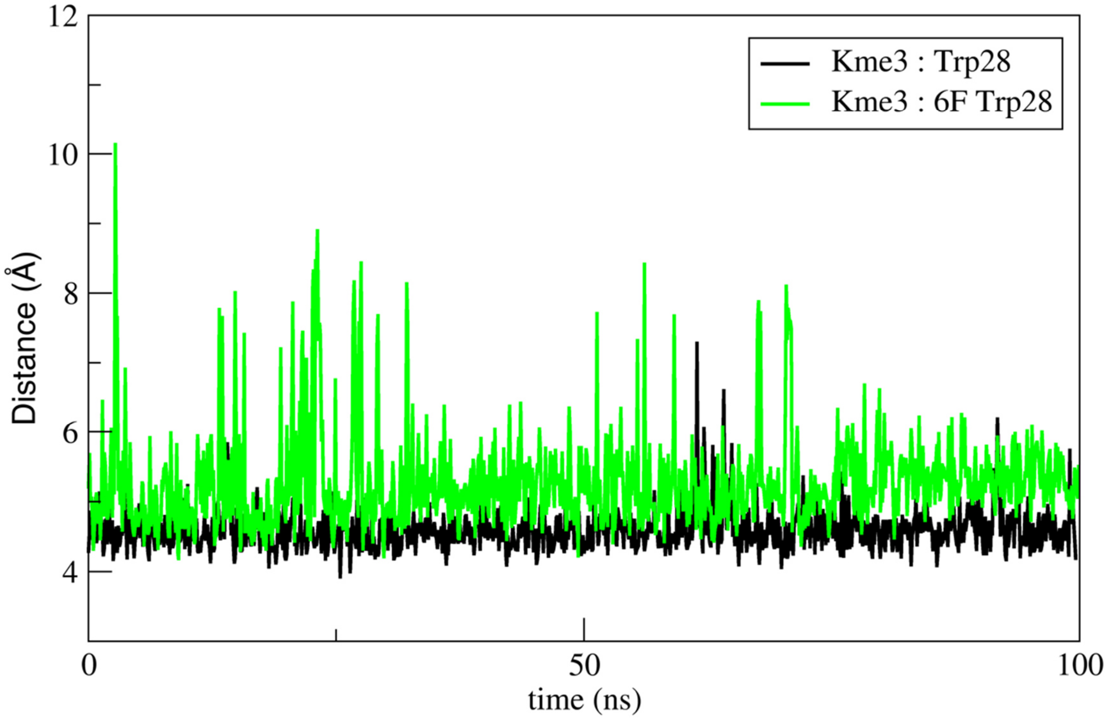

Supplementary Figure 10. Distance vs. time plots of N^+ atom of Kme3 to Trp18, Trp28, 6F-Trp18, and 6F-Trp28 side chain center of mass over 100 ns.

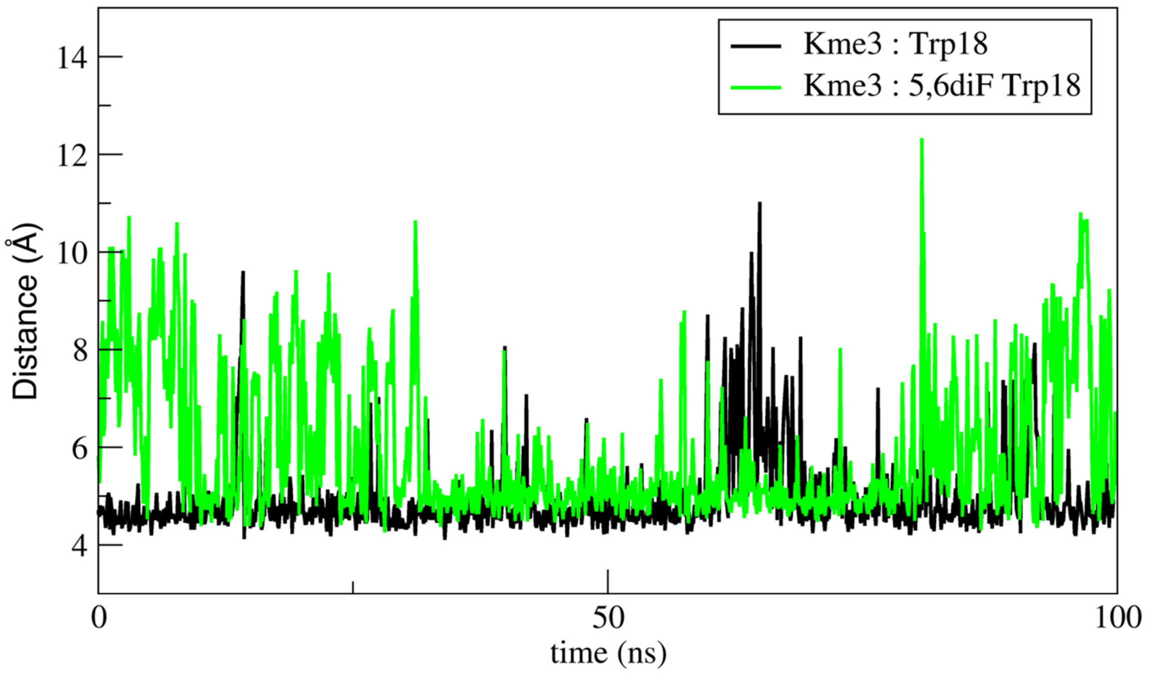

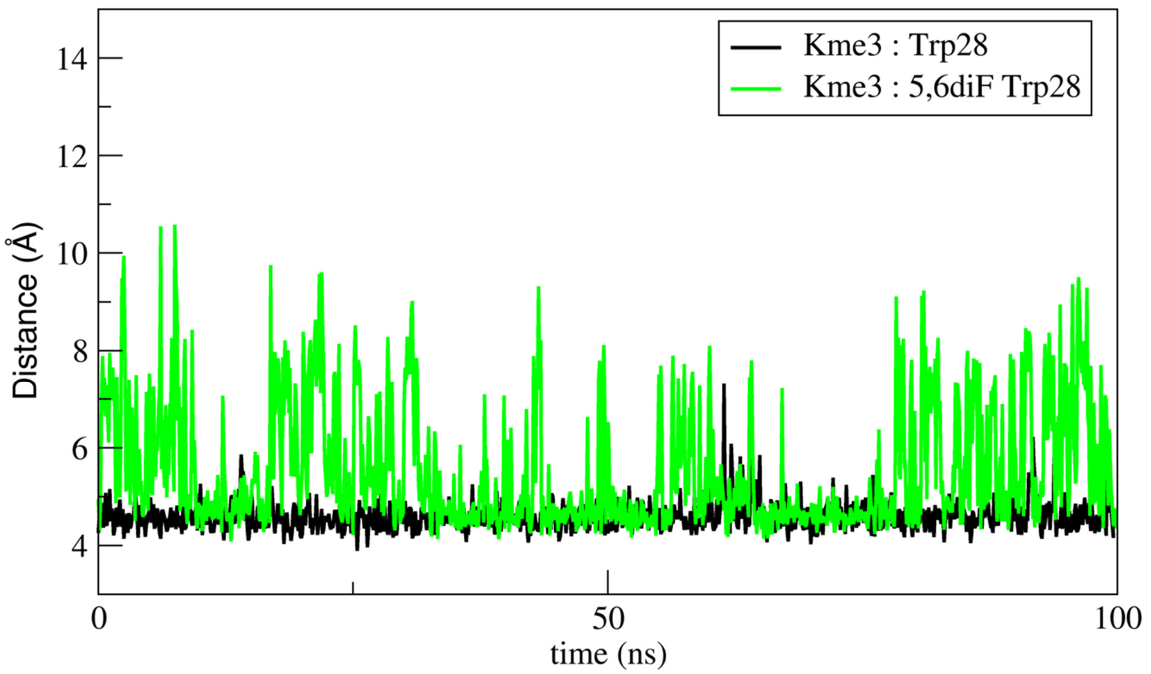

Supplementary Figure 11. Distance vs. time plots of N^+ atom of Kme3 to Trp18, Trp28, 5,6diF-Trp18, and 5,6diF-Trp28 side chain center of mass over 100 ns.

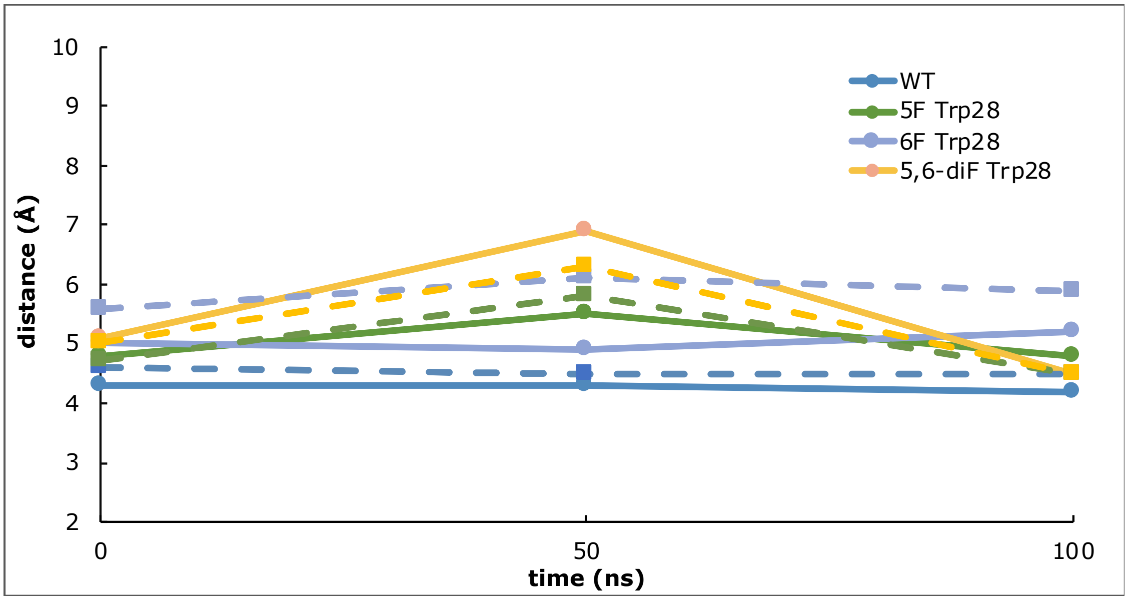

Supplementary Figure 12. Distance calculated from the N^+ atom of H4Kme3 to the 5-membered pyrrole moiety (solid line) and 6-membered benzene moiety (dashed line) of Trp28, 5F-Trp28, 6F-Trp28, and 5,6diF-Trp28 side chains at intervals 0 ns, 50 ns, and 100 ns.

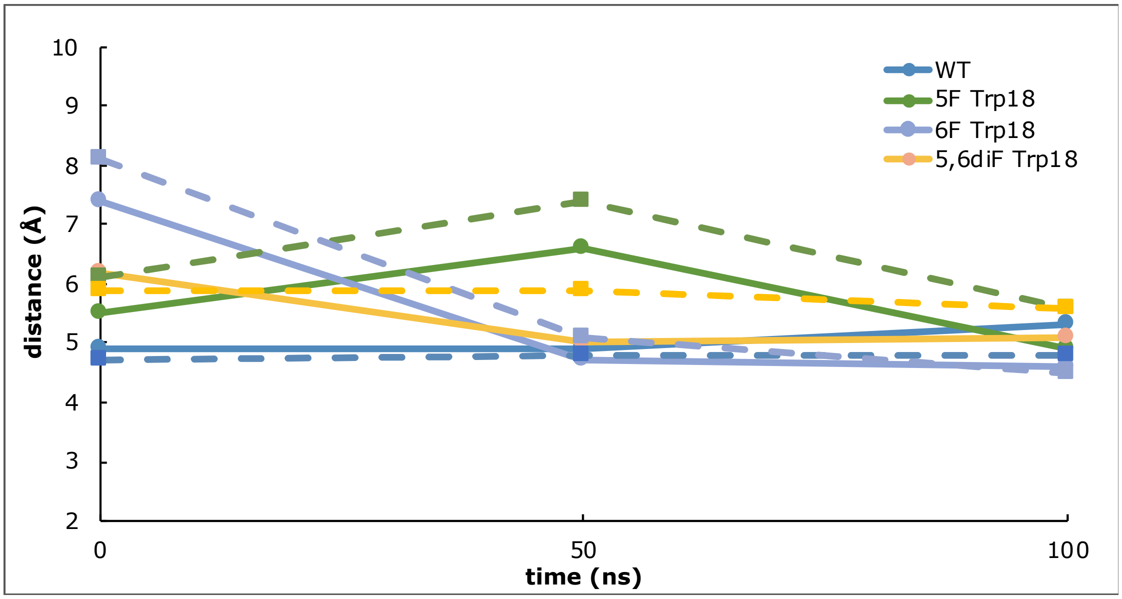

Supplementary Figure 13. Distance calculated from the N^+ atom of H4Kme3 to the 5-membered pyrrole moiety (solid line) and 6-membered benzene moiety (dashed line) of Trp18, 5F-Trp18, 6F-Trp18, and 5,6diF-Trp18 side chains at intervals 0 ns, 50 ns, and 100 ns.

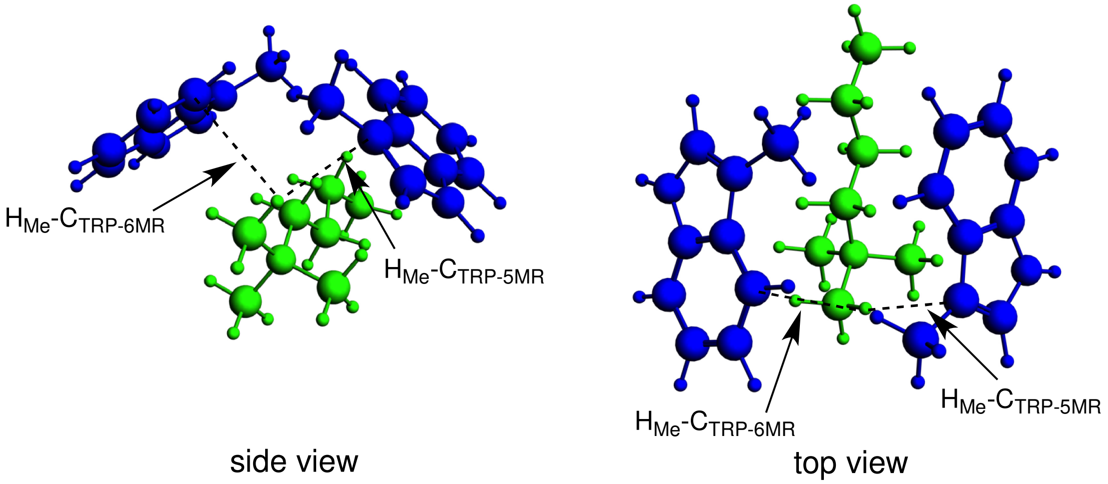

Supplementary Figure 14. Structure of TRP2–Kme3 model complexes. TRP2 in blue and Kme3 in green.

a

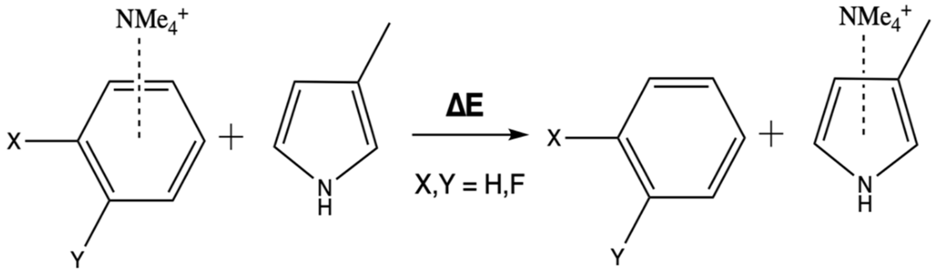

b

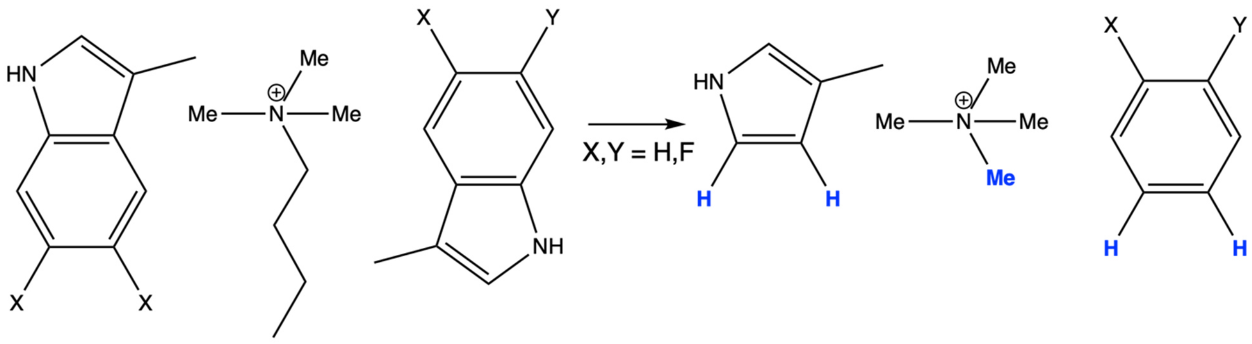

Supplementary Figure 15. (a) Isodemic reaction; **(b)** Structure of TRP2–Kme3 model complexes (left) and model system (right) to analyse separately the interaction between Kme3 and the six- and five-membered rings of TRP2. In blue the atoms that have been added.

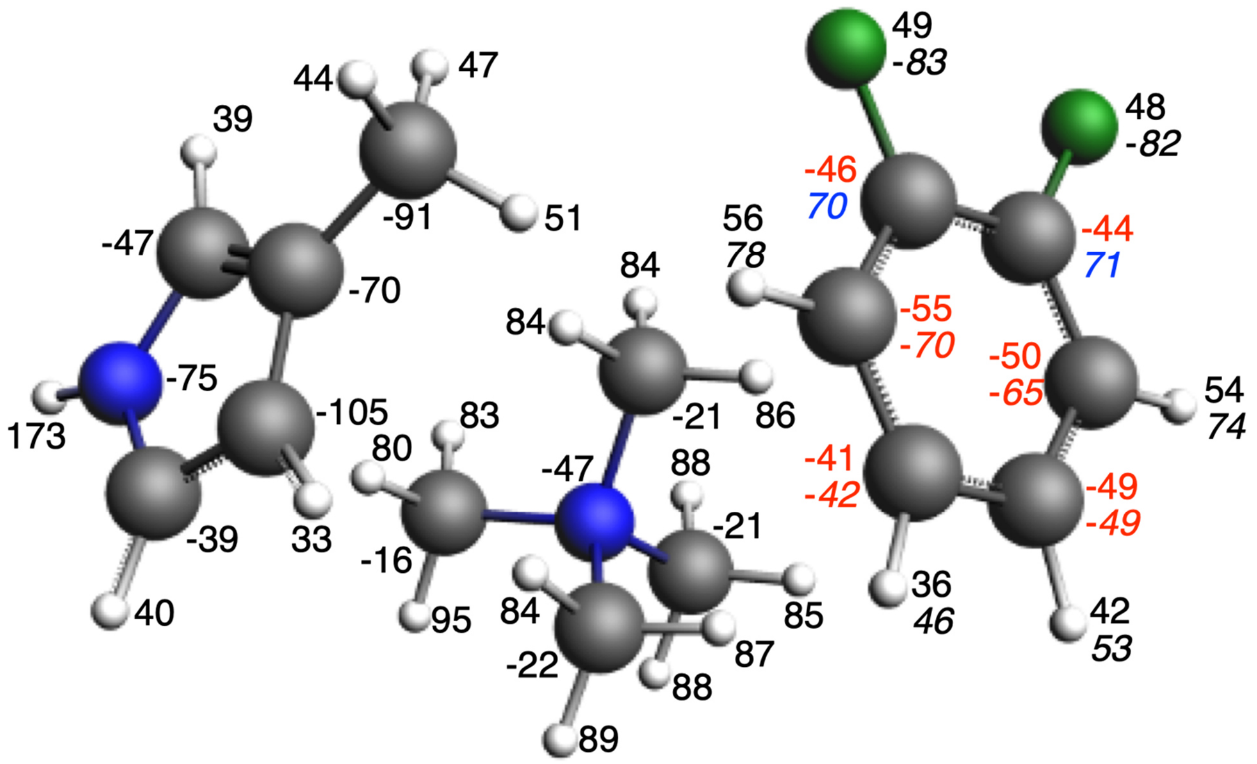

Supplementary Figure 16. VDD atomic charges (in mili-a.u.) in the three separate fragments in the isodesmic reaction in Supplementary Figure 15a. For the six-membered ring, top values are for X, Y = H (derived from Kme3-TRP2), whereas bottom values in italics correspond to X, Y = F (derived from 5,6diF-TRP2). Red (negative) and blue (positive) have been used for the six atoms of the six-membered ring. The VDD values for the five-membered ring (left) and NMe_4^+ (center) correspond to those of X, Y = H (derived from Kme3-TRP2), but only differ in the millesima from those for X, Y = F (derived from 5,6diF-TRP2).

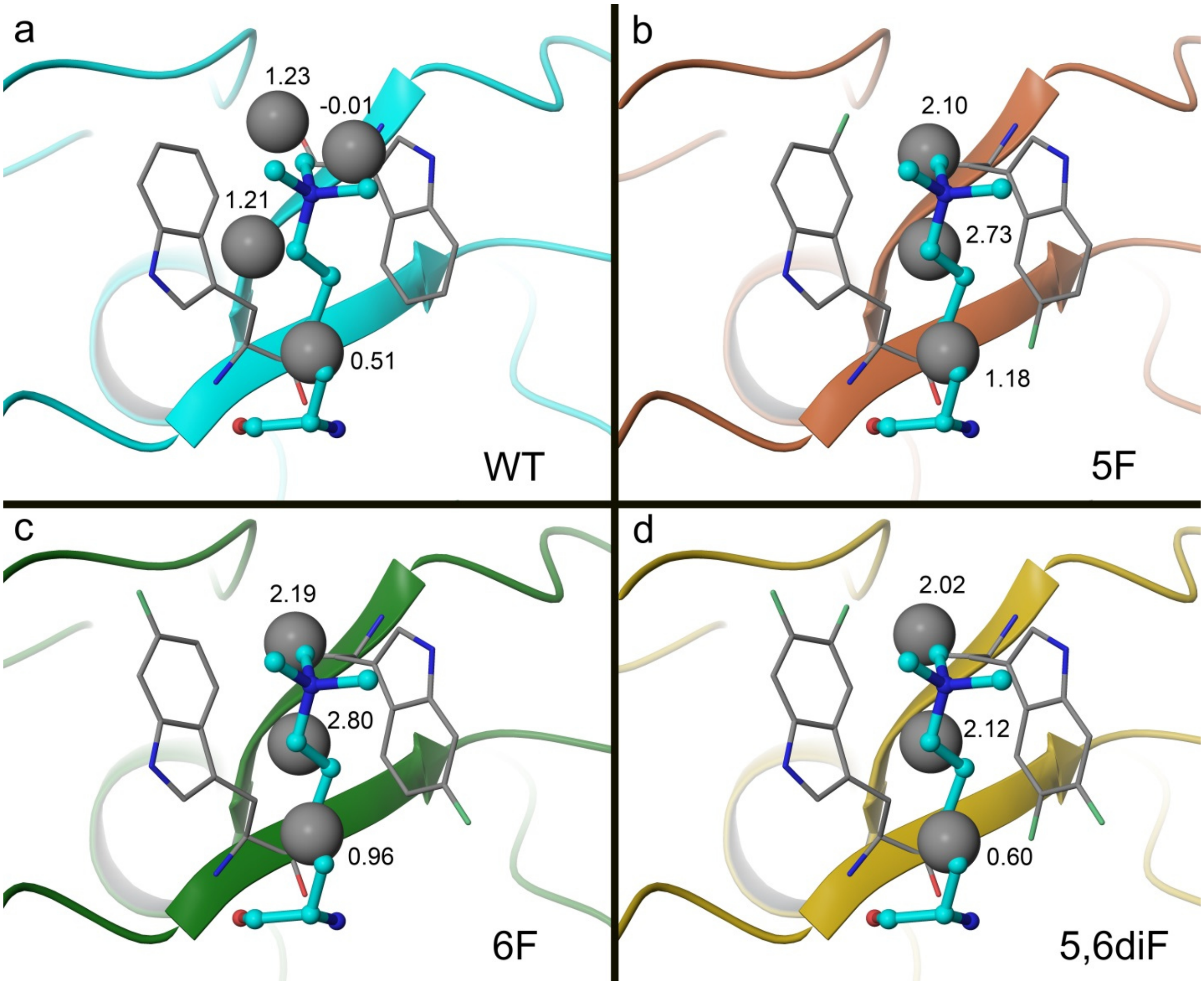

Supplementary Figure 17. Water thermodynamic calculations for the solvation enthalpies of the aromatic cage of KDM5A PHD3 fingers. (**a**) WT-KDM5A; (**b**) 5F-KDM5A; (**c**) 6F-KDM5A; (**d**) 5,6diF-KDM5A. Superimposed Kme3 side chain and water molecules are presented as cyan stick and grey spheres. Numbers adjacent to grey spheres represent the value of the enthalpy (ΔH) for individual water molecule.

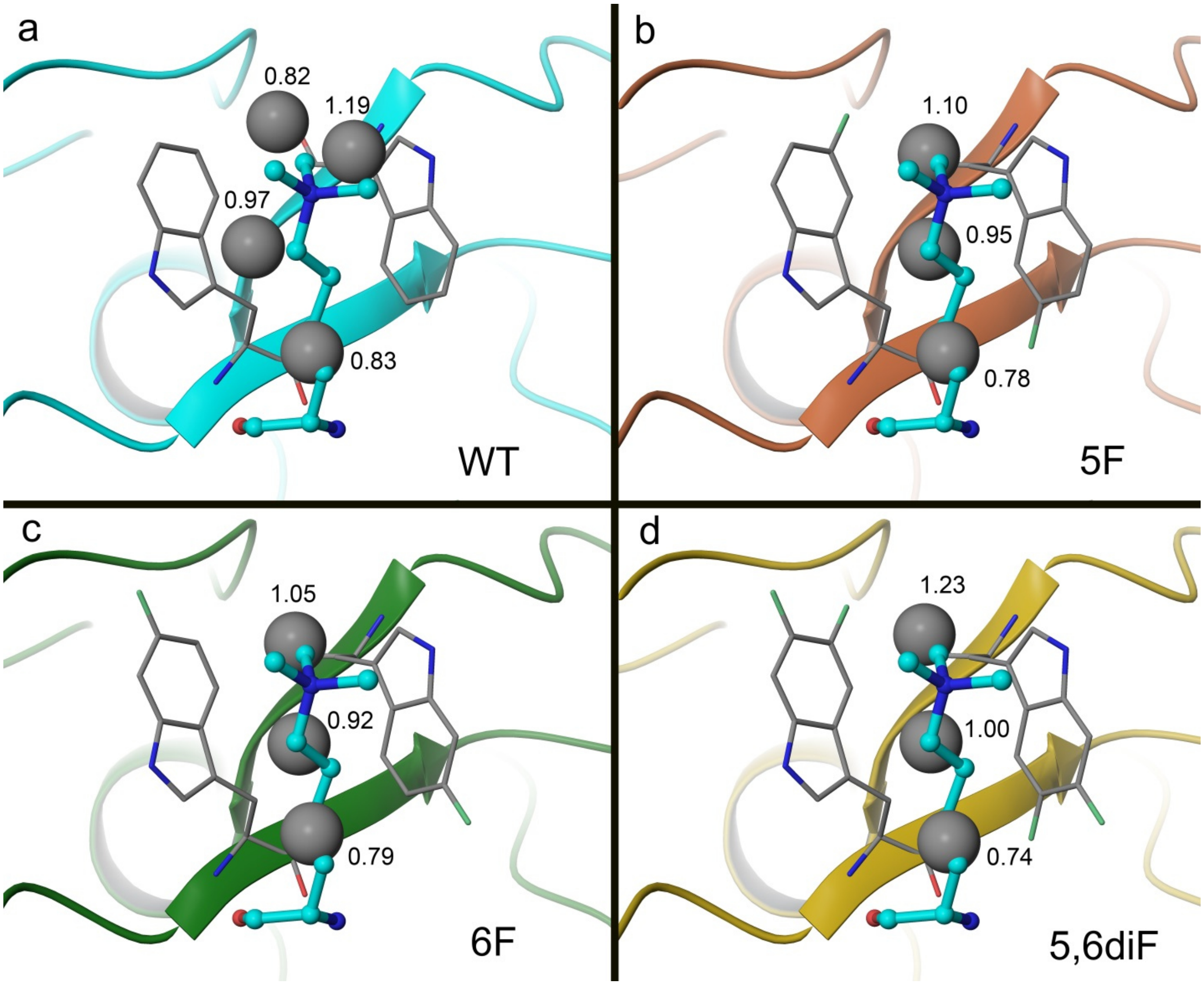

Supplementary Figure 18. Water thermodynamic calculations for the solvation entropies of the aromatic cage of KDM5A PHD3 fingers. (**a**) WT-KDM5A; (**b**) 5F-KDM5A; (**c**) 6F-KDM5A; (**d**) 5,6diF-KDM5A. Superimposed Kme3 side chain and water molecules are presented as cyan stick and grey spheres. Numbers adjacent to grey spheres represent the value of the entropy (–TΔS) for individual water molecule.

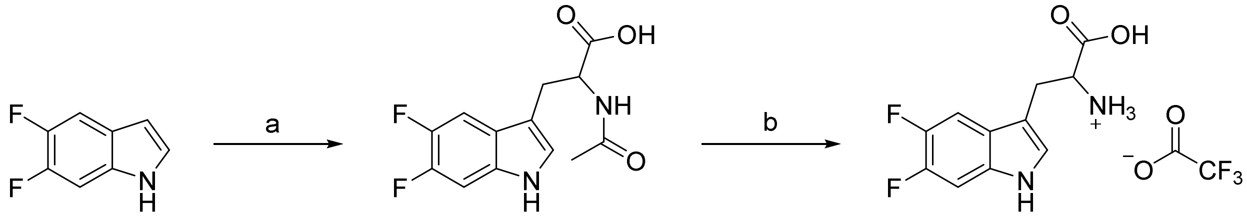

Supplementary Figure 19. Synthetic scheme for the preparation of 5,6-difluorotrypthophan. Conditions: a) L-serine, Ac_2O, AcOH, 70 °C, 74%; b) NaOH, H_2O, 100 °C, 23%.

Supplementary Table 1. Average root mean square deviation (RMSD) and error of non-terminal C_α atoms for each PHD3 KDM5A system.

Protein	RMSD (Å)
**WT-KDM5A**	2.2 ± 0.7
**5F-KDM5A**	2.6 ± 0.5
**6F-KDM5A**	1.7 ± 0.7
**5,6diF-KDM5A**	1.5 ± 0.7

Supplementary Table 2. Average calculated Coulombic electrostatic energy (ΔE_{ele}) (in kcal mol^{-1}) between the terminal N^+ atom of the histone peptide H3K4me3 to the π-system of reader protein aromatic cage residues over 100 ns.

**Residue**	**Indole[a]**	**Indole + F[b]**
**Trp18**	–11.9	-
**Trp28**	–13.1	-
**5F-Trp18**	–6.7	–8.5
**5F-Trp28**	–7.1	–7.8
**6F-Trp18**	–5.2	–5.8
**6F-Trp28**	–7.4	–8.6
**5,6diF-Trp18**	–4.4	–6.0
**5,6diF-Trp28**	–4.0	–6.5

[a] Indole heavy atoms

[b] Indole heavy atoms and electronegative substituents

Supplementary Table 3. (**a**) Cartesian coordinates (in Å) of TRP2–Kme3 systems (with ADF total energies), computed at BLYP-D3BJ/TZ2P using COSMO to simulate aqueous solvation and a constrained optimization to simulate the effect of the protein backbone. (**b**) Cartesian coordinates (in Å) of the fragments of TRP2–Kme3 systems (with ADF total energies), computed at BLYP-D3BJ/TZ2P using COSMO to simulate aqueous solvation.

a) TRP2–Kme3 systems:

TRP2–Kme3 (–8410.6 kcal mol^{-1}):

C	-14.114000000	-20.049000000	-0.875000000
C	-14.962000000	-19.738000000	0.323000000
C	-15.235000000	-20.561000000	1.377000000
C	-15.571000000	-18.476000000	0.628000000
C	-16.191000000	-18.610000000	1.893000000
C	-15.649000000	-17.250000000	-0.044000000
N	-15.971000000	-19.886000000	2.326000000
C	-16.882000000	-17.550000000	2.500000000
C	-16.335000000	-16.198000000	0.561000000
C	-16.943000000	-16.358000000	1.823000000
H	-17.473000000	-15.517000000	2.270000000
H	-14.000000000	-19.128000000	-1.447000000
H	-14.917000000	-21.601000000	1.456000000
H	-15.183000000	-17.121000000	-1.021000000
H	-16.295000000	-20.273000000	3.201000000
H	-17.354000000	-17.669000000	3.475000000
H	-16.402000000	-15.237000000	0.051000000
H	-13.186000000	-20.452000000	-0.470000000
C	-13.008000000	-14.944000000	-1.752000000
C	-11.604000000	-15.279000000	-1.421000000
C	-10.629000000	-14.423000000	-0.994000000
C	-10.999000000	-16.571000000	-1.507000000
C	-9.651000000	-16.428000000	-1.114000000
C	-11.469000000	-17.840000000	-1.880000000
N	-9.451000000	-15.109000000	-0.805000000
C	-8.764000000	-17.507000000	-1.084000000
C	-10.588000000	-18.912000000	-1.851000000
C	-9.247000000	-18.738000000	-1.453000000
H	-8.579000000	-19.599000000	-1.438000000
H	-13.651000000	-15.747000000	-1.391000000
H	-10.764000000	-13.354000000	-0.828000000
H	-12.506000000	-17.981000000	-2.186000000
H	-8.581000000	-14.705000000	-0.490000000

H	-7.726000000	-17.376000000	-0.779000000
H	-10.938000000	-19.903000000	-2.140000000
H	-13.236000000	-13.992000000	-1.272000000
H	-14.522374582	-20.815706247	-1.547885097
H	-13.158423449	-14.818069475	-2.834003080
C	-10.114752602	-21.305220763	1.892377384
C	-11.216790553	-20.285805453	1.565697139
C	-11.002507694	-18.940388922	2.287874794
C	-12.090102444	-17.946438531	1.883398917
N	-12.070579136	-16.609009105	2.645799587
C	-13.150209827	-15.721875154	2.061139245
C	-10.731973677	-15.916856909	2.492497579
C	-12.365551231	-16.823749860	4.115733524
H	-12.408951734	-15.846708144	4.598931529
H	-9.130774938	-20.927740553	1.584711029
H	-12.198761989	-20.691389652	1.844793551
H	-10.013332154	-18.548608026	2.021388165
H	-13.089556823	-18.360771942	2.048366284
H	-12.918787327	-15.550085307	1.009793743
H	-10.798479096	-14.941374934	2.976931329
H	-11.570405162	-17.423783848	4.555007649
H	-10.074709060	-21.512398227	2.969877086
H	-11.238229637	-20.105747697	0.484787617
H	-11.011890000	-19.111207919	3.371339633
H	-11.995495315	-17.683123359	0.825941434
H	-13.153012338	-14.778635478	2.609325685
H	-10.522561113	-15.800602201	1.428638506
H	-13.326128627	-17.334212341	4.203429545
H	-14.111323996	-16.226695834	2.162298263
H	-9.963428736	-16.520728269	2.972373589
H	-10.290718944	-22.253949886	1.371226478

5F-TRP2–Kme3 (–8419.9 kcal mol^{-1}):

C	-14.114000	-20.049000	-0.875000
C	-14.962000	-19.738000	0.323000
C	-15.235000	-20.561000	1.377000
C	-15.571000	-18.476000	0.628000
C	-16.191000	-18.610000	1.893000
C	-15.649000	-17.250000	-0.044000
N	-15.971000	-19.886000	2.326000
C	-16.882000	-17.550000	2.500000
C	-16.335000	-16.198000	0.561000
C	-16.943000	-16.358000	1.823000
H	-17.473000	-15.517000	2.270000
H	-14.000000	-19.128000	-1.447000
H	-14.917000	-21.601000	1.456000
H	-15.183000	-17.121000	-1.021000
H	-16.295000	-20.273000	3.201000
H	-17.354000	-17.669000	3.475000
F	-16.423296	-14.974906	-0.074208
H	-13.186000	-20.452000	-0.470000

C	-13.008000	-14.944000	-1.752000
C	-11.604000	-15.279000	-1.421000
C	-10.629000	-14.423000	-0.994000
C	-10.999000	-16.571000	-1.507000
C	-9.651000	-16.428000	-1.114000
C	-11.469000	-17.840000	-1.880000
N	-9.451000	-15.109000	-0.805000
C	-8.764000	-17.507000	-1.084000
C	-10.588000	-18.912000	-1.851000
C	-9.247000	-18.738000	-1.453000
H	-8.579000	-19.599000	-1.438000
H	-13.651000	-15.747000	-1.391000
H	-10.764000	-13.354000	-0.828000
H	-12.506000	-17.981000	-2.186000
H	-8.581000	-14.705000	-0.490000
H	-7.726000	-17.376000	-0.779000
F	-11.011610	-20.170753	-2.225820
H	-13.236000	-13.992000	-1.272000
H	-14.522375	-20.815706	-1.547885
H	-13.158423	-14.818069	-2.834003
C	-10.114753	-21.305221	1.892377
C	-11.219754	-20.292319	1.562266
C	-11.003437	-18.942821	2.274527
C	-12.083480	-17.945613	1.859024
N	-12.086523	-16.623754	2.646656
C	-13.109805	-15.702360	2.017019
C	-10.728286	-15.954969	2.590006
C	-12.479330	-16.864589	4.090022
H	-12.515032	-15.899948	4.597763
H	-9.132993	-20.928803	1.576224
H	-12.199364	-20.697712	1.849008
H	-10.011026	-18.556615	2.012146
H	-13.085423	-18.365877	1.990196
H	-12.801084	-15.499928	0.990837
H	-10.811843	-14.980917	3.073735
H	-11.737567	-17.508775	4.559551
H	-10.067908	-21.499703	2.971972
H	-11.248217	-20.124883	0.479613
H	-11.018671	-19.107163	3.358799
H	-11.961762	-17.663561	0.809397
H	-13.139750	-14.777949	2.594908
H	-10.443551	-15.837901	1.543107
H	-13.462785	-17.337930	4.102903
H	-14.080886	-16.197561	2.035597
H	-10.005395	-16.574695	3.118021
H	-10.290044	-22.260602	1.383567

6F-TRP2–Kme3 (–8420.6 kcal mol^{-1}):

C	-14.114000	-20.049000	-0.875000
C	-14.962000	-19.738000	0.323000
C	-15.235000	-20.561000	1.377000

C	-15.571000	-18.476000	0.628000
C	-16.191000	-18.610000	1.893000
C	-15.649000	-17.250000	-0.044000
N	-15.971000	-19.886000	2.326000
C	-16.882000	-17.550000	2.500000
C	-16.335000	-16.198000	0.561000
C	-16.943000	-16.358000	1.823000
F	-17.610374	-15.286217	2.381791
H	-14.000000	-19.128000	-1.447000
H	-14.917000	-21.601000	1.456000
H	-15.183000	-17.121000	-1.021000
H	-16.295000	-20.273000	3.201000
H	-17.354000	-17.669000	3.475000
H	-16.402000	-15.237000	0.051000
H	-13.186000	-20.452000	-0.470000
C	-13.008000	-14.944000	-1.752000
C	-11.604000	-15.279000	-1.421000
C	-10.629000	-14.423000	-0.994000
C	-10.999000	-16.571000	-1.507000
C	-9.651000	-16.428000	-1.114000
C	-11.469000	-17.840000	-1.880000
N	-9.451000	-15.109000	-0.805000
C	-8.764000	-17.507000	-1.084000
C	-10.588000	-18.912000	-1.851000
C	-9.247000	-18.738000	-1.453000
F	-8.408961	-19.834104	-1.436774
H	-13.651000	-15.747000	-1.391000
H	-10.764000	-13.354000	-0.828000
H	-12.506000	-17.981000	-2.186000
H	-8.581000	-14.705000	-0.490000
H	-7.726000	-17.376000	-0.779000
H	-10.938000	-19.903000	-2.140000
H	-13.236000	-13.992000	-1.272000
H	-14.522375	-20.815706	-1.547885
H	-13.158423	-14.818069	-2.834003
C	-10.114753	-21.305221	1.892377
C	-11.218530	-20.291317	1.561897
C	-11.006787	-18.944600	2.281166
C	-12.078401	-17.943678	1.853034
N	-12.093365	-16.626090	2.647599
C	-13.097950	-15.696921	1.999726
C	-10.731351	-15.963070	2.624644
C	-12.520167	-16.873829	4.080010
H	-12.558650	-15.912815	4.594367
H	-9.131993	-20.928260	1.579905
H	-12.199501	-20.698025	1.842152
H	-10.009885	-18.560209	2.033025
H	-13.082878	-18.363433	1.964771
H	-12.761747	-15.485802	0.983988
H	-10.820935	-14.991326	3.111993
H	-11.795299	-17.528859	4.560749

H	-10.070841	-21.503560	2.971421
H	-11.241410	-20.116598	0.479972
H	-11.035907	-19.111073	3.364888
H	-11.938778	-17.656218	0.807191
H	-13.142393	-14.777481	2.584911
H	-10.423649	-15.841366	1.584606
H	-13.507924	-17.338262	4.066867
H	-14.070036	-16.189724	1.984958
H	-10.022623	-16.588655	3.164748
H	-10.288614	-22.258907	1.379849

5,6diF-TRP2–Kme3 (–8422.3 kcal mol^{-1}):

C	-14.114000	-20.049000	-0.875000
C	-14.962000	-19.738000	0.323000
C	-15.235000	-20.561000	1.377000
C	-15.571000	-18.476000	0.628000
C	-16.191000	-18.610000	1.893000
C	-15.649000	-17.250000	-0.044000
N	-15.971000	-19.886000	2.326000
C	-16.882000	-17.550000	2.500000
C	-16.335000	-16.198000	0.561000
C	-16.943000	-16.358000	1.823000
F	-17.604394	-15.283210	2.368365
H	-14.000000	-19.128000	-1.447000
H	-14.917000	-21.601000	1.456000
H	-15.183000	-17.121000	-1.021000
H	-16.295000	-20.273000	3.201000
H	-17.354000	-17.669000	3.475000
F	-16.429332	-14.975425	-0.061051
H	-13.186000	-20.452000	-0.470000
C	-13.008000	-14.944000	-1.752000
C	-11.604000	-15.279000	-1.421000
C	-10.629000	-14.423000	-0.994000
C	-10.999000	-16.571000	-1.507000
C	-9.651000	-16.428000	-1.114000
C	-11.469000	-17.840000	-1.880000
N	-9.451000	-15.109000	-0.805000
C	-8.764000	-17.507000	-1.084000
C	-10.588000	-18.912000	-1.851000
C	-9.247000	-18.738000	-1.453000
F	-8.423584	-19.837840	-1.443201
H	-13.651000	-15.747000	-1.391000
H	-10.764000	-13.354000	-0.828000
H	-12.506000	-17.981000	-2.186000
H	-8.581000	-14.705000	-0.490000
H	-7.726000	-17.376000	-0.779000
F	-10.997578	-20.170305	-2.222189
H	-13.236000	-13.992000	-1.272000
H	-14.522375	-20.815706	-1.547885
H	-13.158423	-14.818069	-2.834003

C	-10.114753	-21.305221	1.892377
C	-11.218338	-20.289057	1.566863
C	-10.998781	-18.940937	2.281138
C	-12.077845	-17.941220	1.869066
N	-12.076512	-16.619374	2.656491
C	-13.095134	-15.693896	2.025696
C	-10.715586	-15.955483	2.601323
C	-12.471573	-16.858903	4.099805
H	-12.501205	-15.894390	4.608057
H	-9.132678	-20.931715	1.574051
H	-12.198066	-20.693120	1.854801
H	-10.005728	-18.555329	2.020071
H	-13.079970	-18.360259	2.002932
H	-12.784129	-15.490637	1.000255
H	-10.795460	-14.981873	3.086434
H	-11.734148	-17.508435	4.568655
H	-10.066234	-21.500807	2.971576
H	-11.250938	-20.120838	0.484023
H	-11.013312	-19.107229	3.364978
H	-11.959121	-17.658632	0.819200
H	-13.124979	-14.770286	2.604691
H	-10.429812	-15.837899	1.554799
H	-13.457918	-17.326132	4.111864
H	-14.067149	-16.187530	2.041347
H	-9.995455	-16.578424	3.129055
H	-10.294800	-22.259330	1.383077

b) Fragments of TRP2–Kme3 systems:

Kme3 (fully relaxed, –2976.6 kcal mol^{-1}):

C	-10.159592	-21.338292	1.922272
C	-11.140807	-20.246367	1.464009
C	-11.065384	-18.986679	2.352417
C	-12.063954	-17.941030	1.860819
N	-12.084709	-16.618754	2.648033
C	-13.139016	-15.726591	2.022439
C	-10.742751	-15.919741	2.567204
C	-12.445240	-16.858465	4.100036
H	-12.531633	-15.888682	4.591786
H	-9.124461	-20.974047	1.889441
H	-12.166884	-20.639393	1.480561
H	-10.043702	-18.589397	2.323061
H	-13.086849	-18.324587	1.915082
H	-12.870493	-15.555535	0.979620
H	-10.833526	-14.953293	3.064841
H	-11.658743	-17.447587	4.569182
H	-10.373801	-21.650237	2.952832
H	-10.925840	-19.967545	0.423079
H	-11.282197	-19.268310	3.389846
H	-11.853343	-17.656678	0.825669

H	-13.162713	-14.783904	2.570317
H	-10.487562	-15.784308	1.515147
H	-13.397214	-17.390264	4.135559
H	-14.102836	-16.231883	2.090135
H	-9.993216	-16.530267	3.068242
H	-10.227901	-22.223463	1.279184

TRP2 in TRP2-Kme3 (frozen as in complex, –5423.7 kcal mol^{-1}):

C	-14.114000	-20.049000	-0.875000
C	-14.962000	-19.738000	0.323000
C	-15.235000	-20.561000	1.377000
C	-15.571000	-18.476000	0.628000
C	-16.191000	-18.610000	1.893000
C	-15.649000	-17.250000	-0.044000
N	-15.971000	-19.886000	2.326000
C	-16.882000	-17.550000	2.500000
C	-16.335000	-16.198000	0.561000
C	-16.943000	-16.358000	1.823000
H	-17.473000	-15.517000	2.270000
H	-14.000000	-19.128000	-1.447000
H	-14.917000	-21.601000	1.456000
H	-15.183000	-17.121000	-1.021000
H	-16.295000	-20.273000	3.201000
H	-17.354000	-17.669000	3.475000
H	-16.402000	-15.237000	0.051000
H	-13.186000	-20.452000	-0.470000
C	-13.008000	-14.944000	-1.752000
C	-11.604000	-15.279000	-1.421000
C	-10.629000	-14.423000	-0.994000
C	-10.999000	-16.571000	-1.507000
C	-9.651000	-16.428000	-1.114000
C	-11.469000	-17.840000	-1.880000
N	-9.451000	-15.109000	-0.805000
C	-8.764000	-17.507000	-1.084000
C	-10.588000	-18.912000	-1.851000
C	-9.247000	-18.738000	-1.453000
H	-8.579000	-19.599000	-1.438000
H	-13.651000	-15.747000	-1.391000
H	-10.764000	-13.354000	-0.828000
H	-12.506000	-17.981000	-2.186000
H	-8.581000	-14.705000	-0.490000
H	-7.726000	-17.376000	-0.779000
H	-10.938000	-19.903000	-2.140000
H	-13.236000	-13.992000	-1.272000
H	-14.565798	-20.802079	-1.532639
H	-13.182750	-14.846343	-2.832018

5F-TRP2 in 5F-TRP2–Kme3 (frozen as in complex, –5432.7 kcal mol^{-1}):

C	-14.114000	-20.049000	-0.875000
C	-14.962000	-19.738000	0.323000

C	-15.235000	-20.561000	1.377000
C	-15.571000	-18.476000	0.628000
C	-16.191000	-18.610000	1.893000
C	-15.649000	-17.250000	-0.044000
N	-15.971000	-19.886000	2.326000
C	-16.882000	-17.550000	2.500000
C	-16.335000	-16.198000	0.561000
C	-16.943000	-16.358000	1.823000
H	-17.473000	-15.517000	2.270000
H	-14.000000	-19.128000	-1.447000
H	-14.917000	-21.601000	1.456000
H	-15.183000	-17.121000	-1.021000
H	-16.295000	-20.273000	3.201000
H	-17.354000	-17.669000	3.475000
F	-16.423296	-14.974906	-0.074208
H	-13.186000	-20.452000	-0.470000
C	-13.008000	-14.944000	-1.752000
C	-11.604000	-15.279000	-1.421000
C	-10.629000	-14.423000	-0.994000
C	-10.999000	-16.571000	-1.507000
C	-9.651000	-16.428000	-1.114000
C	-11.469000	-17.840000	-1.880000
N	-9.451000	-15.109000	-0.805000
C	-8.764000	-17.507000	-1.084000
C	-10.588000	-18.912000	-1.851000
C	-9.247000	-18.738000	-1.453000
H	-8.579000	-19.599000	-1.438000
H	-13.651000	-15.747000	-1.391000
H	-10.764000	-13.354000	-0.828000
H	-12.506000	-17.981000	-2.186000
H	-8.581000	-14.705000	-0.490000
H	-7.726000	-17.376000	-0.779000
F	-11.011610	-20.170753	-2.225820
H	-13.236000	-13.992000	-1.272000
H	-14.522375	-20.815706	-1.547885
H	-13.158423	-14.818069	-2.834003

6F-TRP2 in 6F-TRP2–Kme3 (frozen as in complex, –5433.5 kcal mol^{-1}):

C	-14.114000	-20.049000	-0.875000
C	-14.962000	-19.738000	0.323000
C	-15.235000	-20.561000	1.377000
C	-15.571000	-18.476000	0.628000
C	-16.191000	-18.610000	1.893000
C	-15.649000	-17.250000	-0.044000
N	-15.971000	-19.886000	2.326000
C	-16.882000	-17.550000	2.500000
C	-16.335000	-16.198000	0.561000
C	-16.943000	-16.358000	1.823000
F	-17.610374	-15.286217	2.381791
H	-14.000000	-19.128000	-1.447000

H	-14.917000	-21.601000	1.456000
H	-15.183000	-17.121000	-1.021000
H	-16.295000	-20.273000	3.201000
H	-17.354000	-17.669000	3.475000
H	-16.402000	-15.237000	0.051000
H	-13.186000	-20.452000	-0.470000
C	-13.008000	-14.944000	-1.752000
C	-11.604000	-15.279000	-1.421000
C	-10.629000	-14.423000	-0.994000
C	-10.999000	-16.571000	-1.507000
C	-9.651000	-16.428000	-1.114000
C	-11.469000	-17.840000	-1.880000
N	-9.451000	-15.109000	-0.805000
C	-8.764000	-17.507000	-1.084000
C	-10.588000	-18.912000	-1.851000
C	-9.247000	-18.738000	-1.453000
F	-8.408961	-19.834104	-1.436774
H	-13.651000	-15.747000	-1.391000
H	-10.764000	-13.354000	-0.828000
H	-12.506000	-17.981000	-2.186000
H	-8.581000	-14.705000	-0.490000
H	-7.726000	-17.376000	-0.779000
H	-10.938000	-19.903000	-2.140000
H	-13.236000	-13.992000	-1.272000
H	-14.522375	-20.815706	-1.547885
H	-13.158423	-14.818069	-2.834003

5,6diF-TRP2 in 5,6diF-TRP2–Kme3 (frozen as in complex, –5435.2 kcal mol^{-1}):

C	-14.114000	-20.049000	-0.875000
C	-14.962000	-19.738000	0.323000
C	-15.235000	-20.561000	1.377000
C	-15.571000	-18.476000	0.628000
C	-16.191000	-18.610000	1.893000
C	-15.649000	-17.250000	-0.044000
N	-15.971000	-19.886000	2.326000
C	-16.882000	-17.550000	2.500000
C	-16.335000	-16.198000	0.561000
C	-16.943000	-16.358000	1.823000
F	-17.604394	-15.283210	2.368365
H	-14.000000	-19.128000	-1.447000
H	-14.917000	-21.601000	1.456000
H	-15.183000	-17.121000	-1.021000
H	-16.295000	-20.273000	3.201000
H	-17.354000	-17.669000	3.475000
F	-16.429332	-14.975425	-0.061051
H	-13.186000	-20.452000	-0.470000
C	-13.008000	-14.944000	-1.752000
C	-11.604000	-15.279000	-1.421000
C	-10.629000	-14.423000	-0.994000
C	-10.999000	-16.571000	-1.507000

C -9.651000 -16.428000 -1.114000
C -11.469000 -17.840000 -1.880000
N -9.451000 -15.109000 -0.805000
C -8.764000 -17.507000 -1.084000
C -10.588000 -18.912000 -1.851000
C -9.247000 -18.738000 -1.453000
F -8.423584 -19.837840 -1.443201
H -13.651000 -15.747000 -1.391000
H -10.764000 -13.354000 -0.828000
H -12.506000 -17.981000 -2.186000
H -8.581000 -14.705000 -0.490000
H -7.726000 -17.376000 -0.779000
F -10.997578 -20.170305 -2.222189
H -13.236000 -13.992000 -1.272000
H -14.522375 -20.815706 -1.547885
H -13.158423 -14.818069 -2.834003

Kme3 in TRP2–Kme3 (frozen as in complex, –2976.6 kcal mol^{-1}):
C -10.233000 -21.229000 1.628000
C -11.342111 -20.186362 1.456881
C -11.066279 -18.873967 2.216037
C -12.141178 -17.850758 1.851428
N -12.112205 -16.543234 2.659310
C -13.134043 -15.600426 2.058014
C -10.746209 -15.892735 2.589987
C -12.483879 -16.802818 4.104348
H -12.491555 -15.847670 4.631299
H -9.276797 -20.846563 1.247013
H -12.303030 -20.598157 1.792434
H -10.075165 -18.498577 1.933639
H -13.145364 -18.260490 1.996415
H -12.833863 -15.382358 1.032572
H -10.807758 -14.925063 3.089836
H -11.746174 -17.470184 4.547712
H -10.091834 -21.493304 2.684701
H -11.444212 -19.951050 0.393209
H -11.054120 -19.071315 3.295038
H -12.038473 -17.552398 0.804435
H -13.148853 -14.686657 2.653193
H -10.477557 -15.763712 1.540241
H -13.475897 -17.257610 4.126332
H -14.110461 -16.085565 2.073748
H -10.023446 -16.531027 3.096031
H -10.470674 -22.148290 1.079142

Kme3 in 5F-TRP2–Kme3 (frozen as in complex, –2976.5 kcal mol^{-1}):
C -10.114753 -21.305221 1.892377
C -11.219754 -20.292319 1.562266
C -11.003437 -18.942821 2.274527
C -12.083480 -17.945613 1.859024

N	-12.086523	-16.623754	2.646656
C	-13.109805	-15.702360	2.017019
C	-10.728286	-15.954969	2.590006
C	-12.479330	-16.864589	4.090022
H	-12.515032	-15.899948	4.597763
H	-9.132993	-20.928803	1.576224
H	-12.199364	-20.697712	1.849008
H	-10.011026	-18.556615	2.012146
H	-13.085423	-18.365877	1.990196
H	-12.801084	-15.499928	0.990837
H	-10.811843	-14.980917	3.073735
H	-11.737567	-17.508775	4.559551
H	-10.067908	-21.499703	2.971972
H	-11.248217	-20.124883	0.479613
H	-11.018671	-19.107163	3.358799
H	-11.961762	-17.663561	0.809397
H	-13.139750	-14.777949	2.594908
H	-10.443551	-15.837901	1.543107
H	-13.462785	-17.337930	4.102903
H	-14.080886	-16.197561	2.035597
H	-10.005395	-16.574695	3.118021
H	-10.290044	-22.260602	1.383567

Kme3 in 6F-TRP2–Kme3 (frozen as in complex, –2976.5 kcal mol^{-1}):

C	-10.114753	-21.305221	1.892377
C	-11.218530	-20.291317	1.561897
C	-11.006787	-18.944600	2.281166
C	-12.078401	-17.943678	1.853034
N	-12.093365	-16.626090	2.647599
C	-13.097950	-15.696921	1.999726
C	-10.731351	-15.963070	2.624644
C	-12.520167	-16.873829	4.080010
H	-12.558650	-15.912815	4.594367
H	-9.131993	-20.928260	1.579905
H	-12.199501	-20.698025	1.842152
H	-10.009885	-18.560209	2.033025
H	-13.082878	-18.363433	1.964771
H	-12.761747	-15.485802	0.983988
H	-10.820935	-14.991326	3.111993
H	-11.795299	-17.528859	4.560749
H	-10.070841	-21.503560	2.971421
H	-11.241410	-20.116598	0.479972
H	-11.035907	-19.111073	3.364888
H	-11.938778	-17.656218	0.807191
H	-13.142393	-14.777481	2.584911
H	-10.423649	-15.841366	1.584606
H	-13.507924	-17.338262	4.066867
H	-14.070036	-16.189724	1.984958
H	-10.022623	-16.588655	3.164748
H	-10.288614	-22.258907	1.379849

Kme3 in 5,6diF-TRP2–Kme3 (frozen as in complex, –2976.5 kcal mol^{-1}):

C	-10.114753	-21.305221	1.892377
C	-11.218338	-20.289057	1.566863
C	-10.998781	-18.940937	2.281138
C	-12.077845	-17.941220	1.869066
N	-12.076512	-16.619374	2.656491
C	-13.095134	-15.693896	2.025696
C	-10.715586	-15.955483	2.601323
C	-12.471573	-16.858903	4.099805
H	-12.501205	-15.894390	4.608057
H	-9.132678	-20.931715	1.574051
H	-12.198066	-20.693120	1.854801
H	-10.005728	-18.555329	2.020071
H	-13.079970	-18.360259	2.002932
H	-12.784129	-15.490637	1.000255
H	-10.795460	-14.981873	3.086434
H	-11.734148	-17.508435	4.568655
H	-10.066234	-21.500807	2.971576
H	-11.250938	-20.120838	0.484023
H	-11.013312	-19.107229	3.364978
H	-11.959121	-17.658632	0.819200
H	-13.124979	-14.770286	2.604691
H	-10.429812	-15.837899	1.554799
H	-13.457918	-17.326132	4.111864
H	-14.067149	-16.187530	2.041347
H	-9.995455	-16.578424	3.129055
H	-10.294800	-22.259330	1.383077

Supplementary Table 4. Distances (in Å) between the quaternary N atom and the centroids of the five- and six-membered rings of the systems under analysis.

	**TRP2–Kme3**	**5F-TRP2–Kme3**	**6F-TRP2–Kme3**	**5,6diF-TRP2–Kme3**
r_{6a}	4.496	4.479	4.473	4.492
r_{5a}	4.716	4.695	4.689	4.708
r_{5b}	4.268	4.278	4.282	4.282
r_{6b}	4.686	4.690	4.693	4.695

[a] Computed at BLYP-D3BJ/TZ2P.

Supplementary Table 5. Overlaps between the MOs of TRP and Kme3.[a]

TRP MOs	Kme3 MOs	TRP2–Kme3	5F-TRP2–Kme3	6F-TRP2–Kme3	5,6diF-TRP2–Kme3
HOMO	LUMO	0.012	0.009	0.015	0.011
HOMO	LUMO+1	0.006	0.006	0.007	0.009
HOMO-1	LUMO	0.028	0.031	0.023	0.028
HOMO-1	LUMO+1	0.006	0.001	0.010	0.004

[a] Computed at BLYP-D3BJ/TZ2P.

Supplementary Table 6. Isodesmic reaction energies and energy decomposition analysis for the four model systems (values in kcal mol^{-1}).[a]

model	TRP2–Kme3	5F-TRP2–Kme3	6F-TRP2–Kme3	5,6diF-TRP2–Kme3
**ΔE (isodesmic)**	-1.30	-1.52	-1.48	-1.77
**6-MR···NMe_4^+**				
**ΔE_{Pauli}**	6.01	5.97	6.26	5.67
**ΔV_{elstat}**	-5.15	-3.54	-3.48	-1.74
**ΔE_{oi}**	-4.13	-4.18	-4.29	-4.09
**ΔE_{disp}**	-5.36	-5.40	-5.40	-5.34
**ΔE_{int}**	-8.62	-7.16	-6.90	-5.50
**5-MR···NMe_4^+**				
**ΔE_{Pauli}**	7.58	7.46	7.48	7.36
**ΔV_{elstat}**	-7.70	-7.68	-7.70	-7.63
**ΔE_{oi}**	-5.72	-5.69	-5.71	-5.65
**ΔE_{disp}**	-6.17	-6.11	-6.10	-6.08
**ΔE_{int}**	-12.02	-12.03	-12.02	-12.00

[a] Computed at BLYP-D3BJ/TZ2P. Isodemic reaction energies computed in COSMO (aqueous solution), whereas EDA performed in the gas phase.

Supplementary Table 7. Water thermodynamic calculations of thermodynamic parameters (in kcal mol^{-1}) for the solvation of aromatic cages of wild-type and fluorinated PHD3 fingers of KDM5A.

**Protein**	**Δ*G***	**Δ*H***	**–TΔ*S***
**WT-KDM5A**	4.9	1.8	3.1
**5F-KDM5A**	8.0	5.4	2.6
**6F-KDM5A**	7.8	5.3	2.5
**5,6diF-KDM5A**	6.6	4.0	2.6

Supplementary Table 8. Cartesian coordinates and charges calculated using the RESP method HF/6-31G* of modified 5F-Trp residues used in MD simulations.

5F-Trp Atom	X	Y	Z	RESP Charge
N	2.808	0.398	1.397	-1.084269
C	2.418	-0.492	0.332	0.504578
C	3.635	-1.220	-0.195	0.419917
O	4.751	-0.835	-0.062	-0.478103
C	1.721	0.180	-0.882	-0.424242
C	0.423	0.849	-0.533	0.100781
C	0.205	2.177	-0.395	-0.242948
C	-0.847	0.210	-0.268	0.087437
N	-1.099	2.422	-0.059	-0.375718
C	-1.766	1.226	0.021	0.094860
C	-1.274	-1.124	-0.265	-0.349371
C	-3.099	0.954	0.318	-0.210229
C	-2.585	-1.368	0.027	0.362621
C	-3.505	-0.359	0.320	-0.301287
H	3.639	0.900	1.154	0.393316
H	1.742	-1.242	0.735	-0.00983
H	2.405	0.911	-1.304	0.113947
H	1.546	-0.565	-1.656	0.113947
H	0.893	2.989	-0.522	0.210990
H	-1.499	3.320	0.073	0.369296

H	-0.615	-1.943	-0.485	0.179767
H	-3.796	1.743	0.539	0.176683
H	-4.519	-0.631	0.541	0.191751
F	-3.025	-2.629	0.037	-0.223847
H	2.079	1.050	1.608	0.393316
H	3.424	-2.128	-0.769	-0.013363

Supplementary Table 9. Cartesian coordinates and charges calculated using the RESP method HF/6-31G* of modified 6F-Trp residues used in MD simulations.

6F-Trp Atom	X	Y	Z	RESP Charge
N	2.879	0.357	1.445	-1.077819
C	2.568	-0.511	0.338	0.486763
C	3.837	-1.154	-0.178	0.431700
O	4.927	-0.720	0.007	-0.481065
C	1.871	0.168	-0.873	-0.406804
C	0.530	0.753	-0.539	0.112672
C	0.237	2.058	-0.364	-0.266157
C	-0.712	0.034	-0.328	-0.019942
N	-1.094	2.217	-0.055	-0.373504
C	-1.693	0.990	-0.032	0.199442
C	-1.077	-1.315	-0.377	-0.103281
C	-3.021	0.648	0.223	-0.445644
C	-2.380	-1.675	-0.131	-0.389942
C	-3.319	-0.685	0.164	0.437374
H	3.691	0.907	1.249	0.392747
H	1.922	-1.311	0.692	-0.010470
H	2.526	0.950	-1.246	0.109310
H	1.759	-0.555	-1.678	0.109310
H	0.878	2.913	-0.446	0.213168
H	-1.548	3.085	0.099	0.372937

H	-0.351	-2.073	-0.608	0.144826
H	-3.782	1.371	0.451	0.212242
H	-2.699	-2.700	-0.159	0.200781
F	-4.573	-1.068	0.398	-0.224650
H	2.112	0.965	1.653	0.392747
H	3.691	-2.048	-0.794	-0.016740

Supplementary Table 10. Cartesian coordinates and charges calculated using the RESP method HF/6-31G* of modified 5,6diF-Trp residues used in MD simulations.

5,6diF-Trp Atom	X	Y	Z	RESP Charge
N	3.118	0.315	1.436	-1.086308
C	2.722	-0.547	0.351	0.503447
C	3.926	-1.311	-0.158	0.426946
O	5.051	-0.967	0.007	-0.478144
C	2.080	0.164	-0.871	-0.463851
C	0.799	0.875	-0.545	0.186917
C	0.626	2.207	-0.395	-0.321047
C	-0.5	0.279	-0.318	-0.001369
N	-0.681	2.493	-0.086	-0.304664
C	-1.388	1.324	-0.038	0.109248
C	-0.979	-1.037	-0.342	-0.309149
C	-2.741	1.103	0.225	-0.392988
C	-2.302	-1.251	-0.086	0.218428
C	-3.170	-0.192	0.195	0.274993
H	3.975	0.784	1.223	0.395594
H	2.012	-1.279	0.727	-0.009224
H	2.799	0.876	-1.267	0.122537
H	1.897	-0.564	-1.66	0.122537
H	1.342	2.997	-0.494	0.225782
H	-1.053	3.402	0.051	0.358256

H	-0.346	-1.878	-0.557	0.193691
H	-3.434	1.894	0.442	0.223891
F	-4.442	-0.476	0.435	-0.187665
F	-2.803	-2.481	-0.098	-0.188765
H	2.411	0.993	1.636	0.395594
H	3.698	-2.205	-0.748	-0.014687
